# Supplementary figures and images for: SEA CDM: Study-Experiment-Assay Common Data Model and Databases for Cross-Domain Data Integration and Analysis
Source: bioRxiv. 2025 Aug 28:2025.08.26.671804. Preprint. [Version 1] doi: 10.1101/2025.08.26.671804 (PMC12407955; doi:10.1101/2025.08.26.671804)

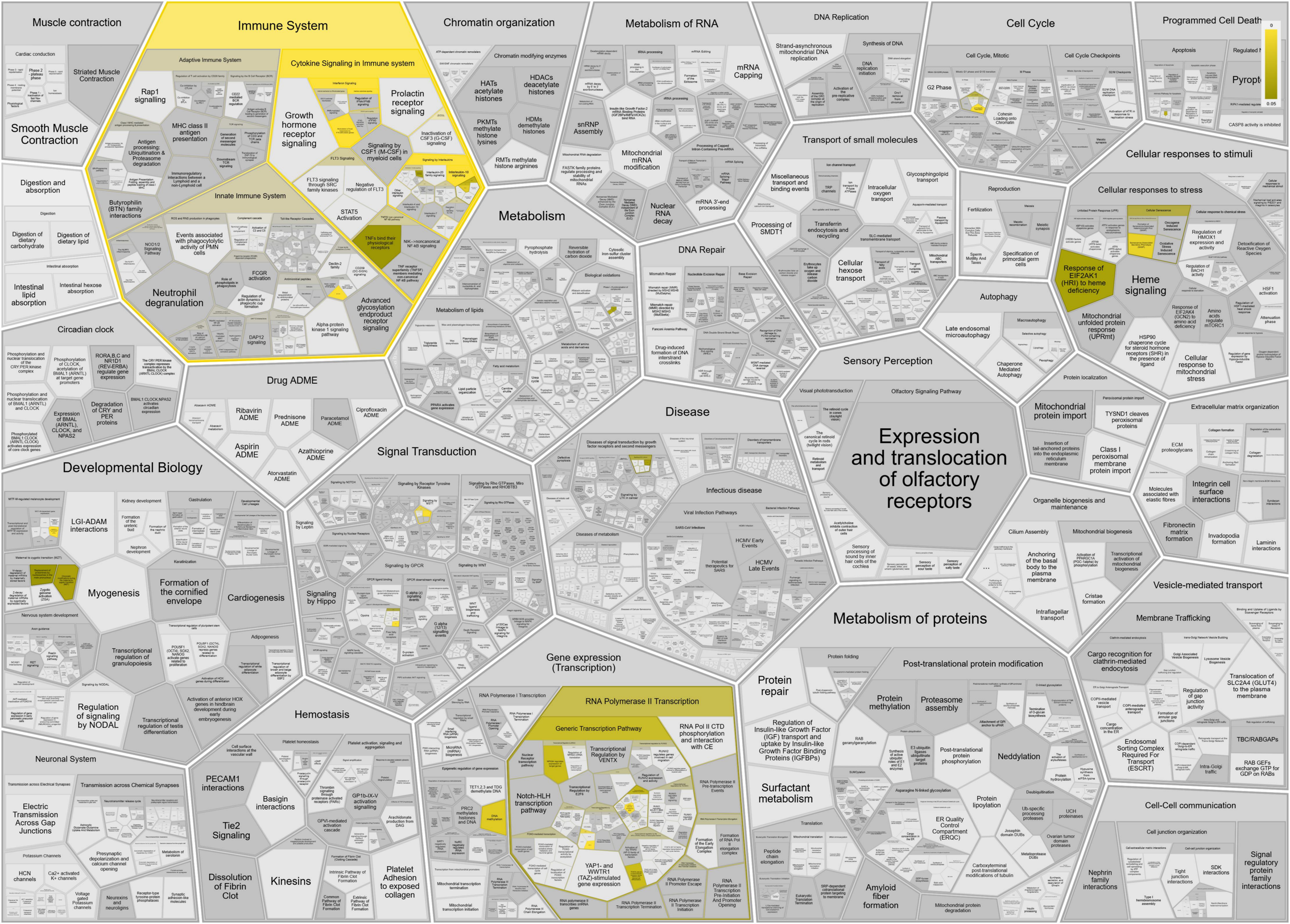

Supplement: Supplement 2 — Supplemental Figure 2. Full Reactome representation of Reactome pathways stimulated by all Influenza vaccines in female human subjects. Gene set enrichment values can be found as part of Supplemental File 2. [file media-2.pdf]

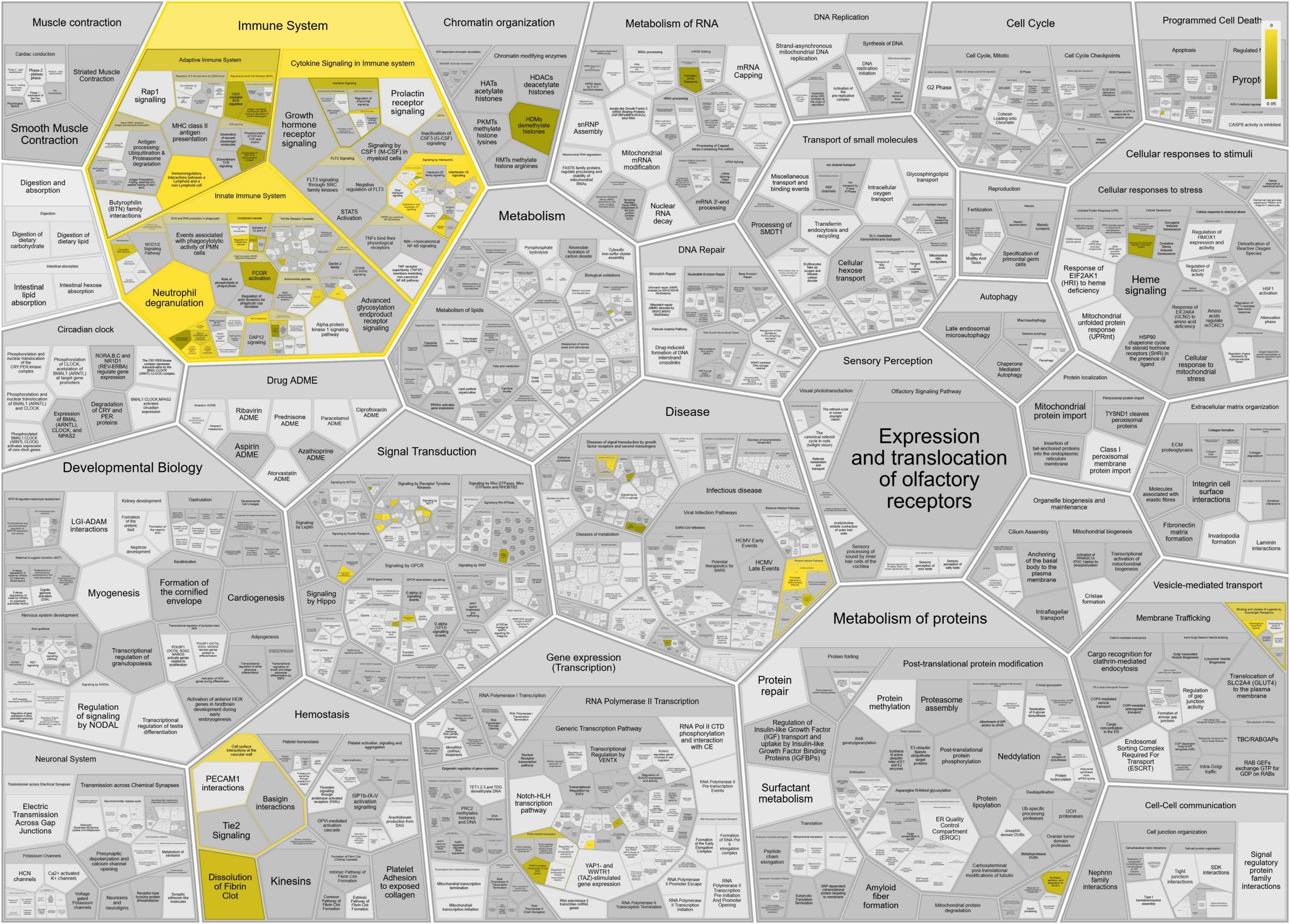

Supplement: Supplement 3 — Supplemental Figure 3. Full Reactome representation of Reactome pathways stimulated by all Influenza vaccines in male human subjects. Gene set enrichment values can be found as part of Supplemental File 2. [file media-3.pdf]

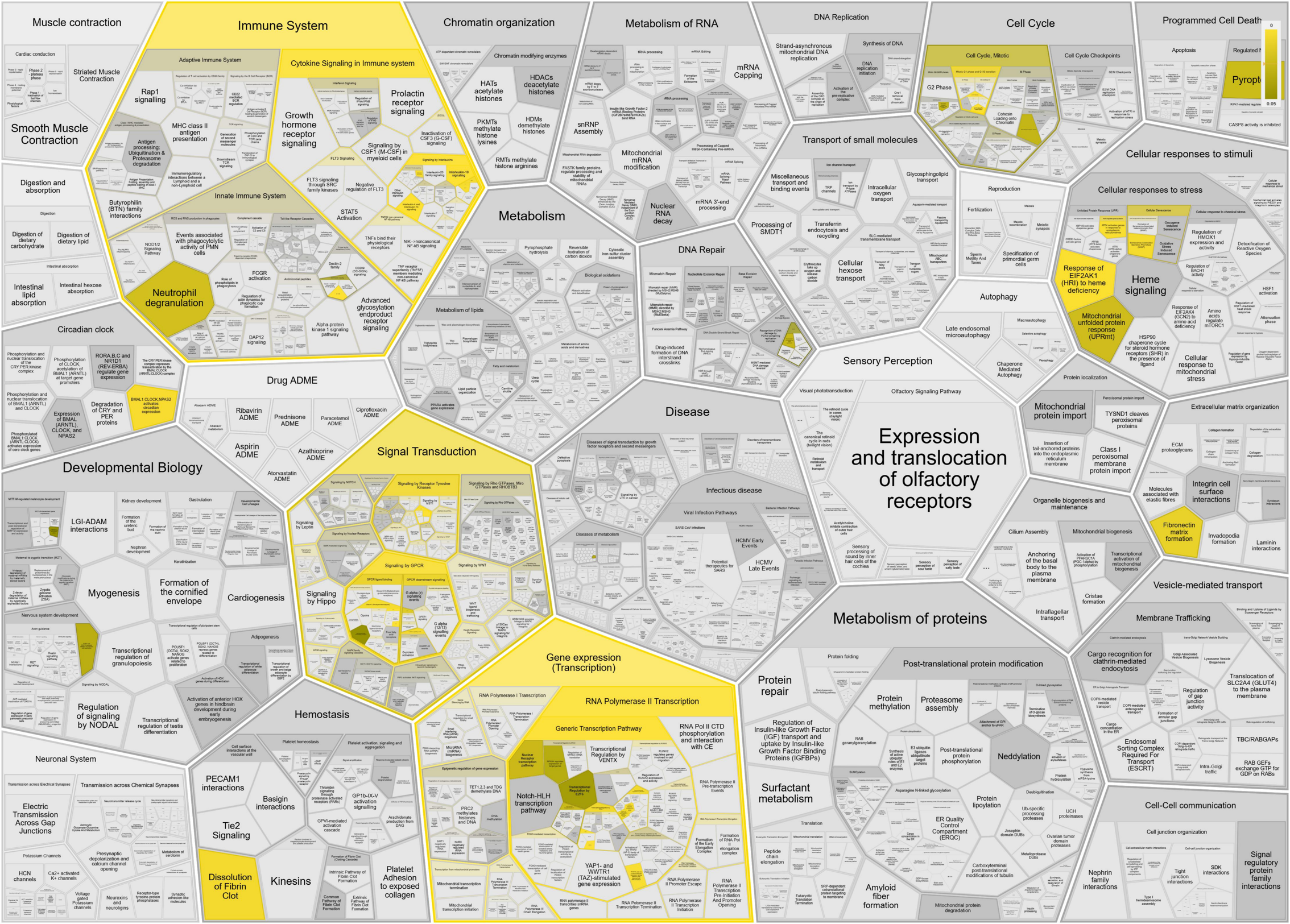

Supplement: Supplement 5 — Supplemental Figure 5. Full Reactome representation of Reactome pathways stimulated by all Influenza and live attenuated Influenza vaccines in female human subjects. Gene set enrichment values can be found as part of Supplemental File 2. [file media-5.pdf]

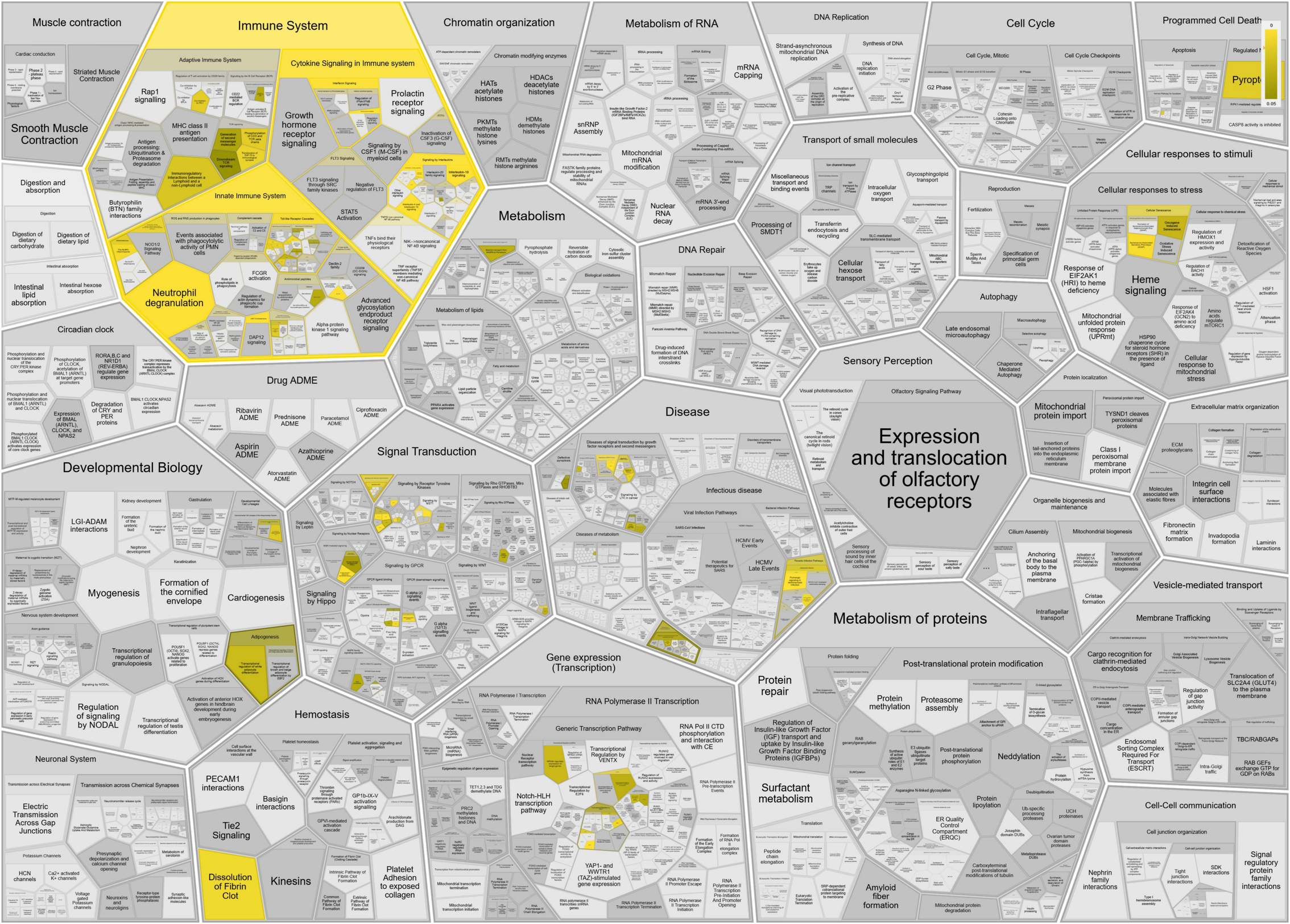

Supplement: Supplement 9 — Supplemental Figure 9. Full Reactome representation of Reactome pathways stimulated by trivalent inactivated Influenza vaccines in male human subjects. Gene set enrichment values can be found as part of Supplemental File 2. [file media-9.pdf]

KEGG Subset Comparison (All) – Top 10

Pathway name

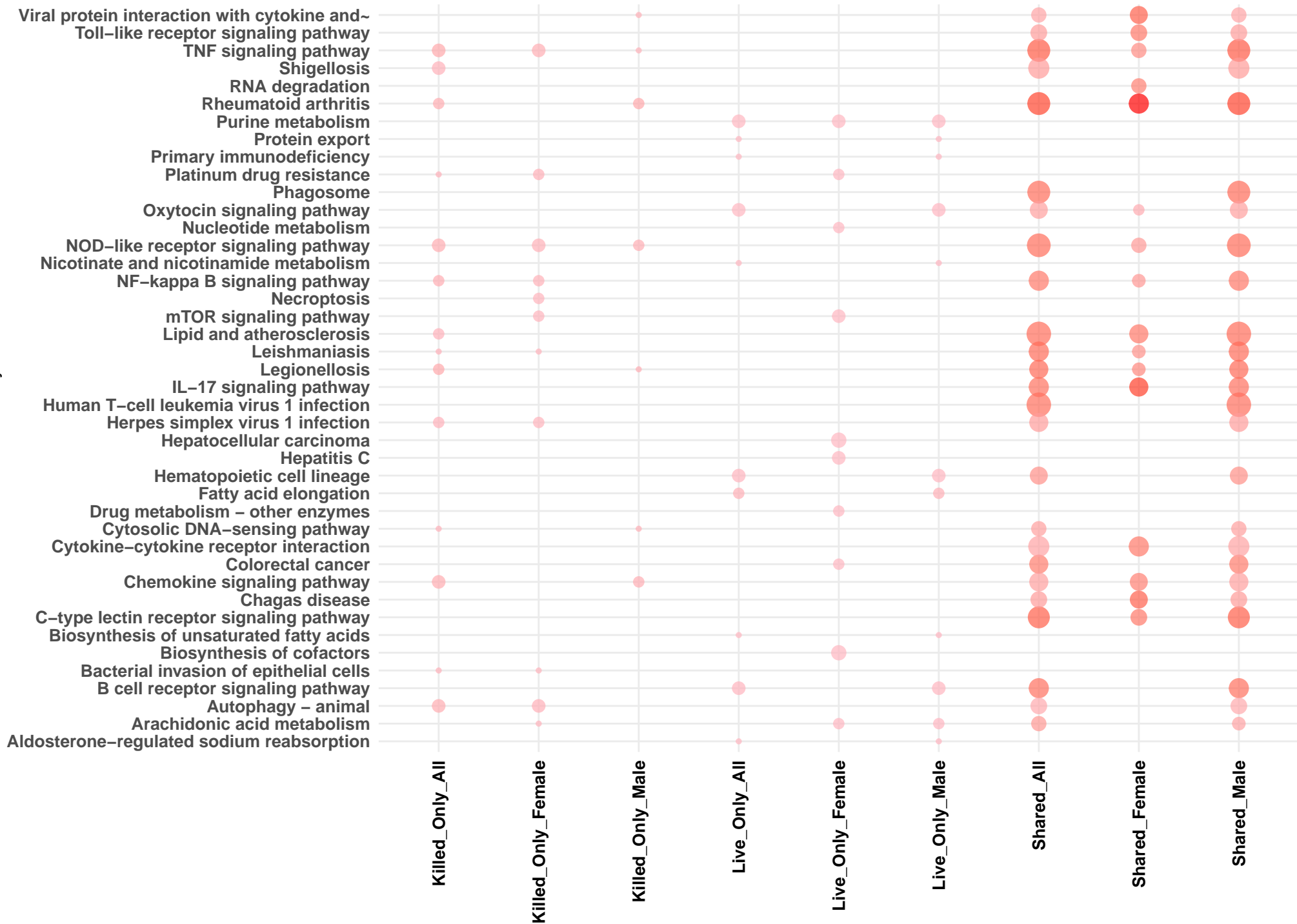

Supplement: Supplement 10 — Supplemental Figure 10. GO functional analysis results of influenza vaccines. Any pathway listed shows up as one of the top 10 most significant GO pathways for one of the nine gene sets. [file media-10.pdf]

# KEGG Subset Comparison (All) – Top 10

Pathway name

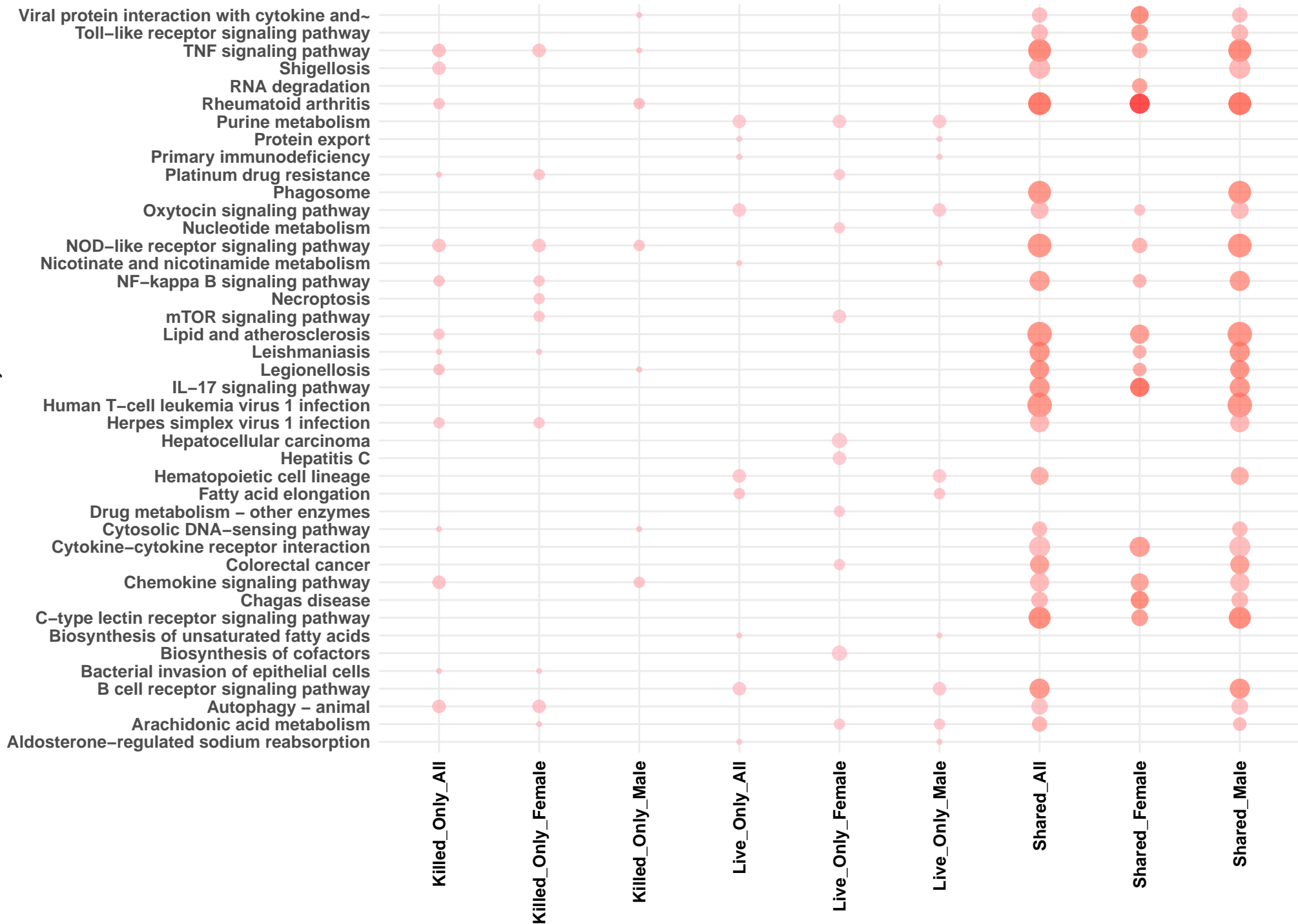

$-\log_{10}(\text{Padj})$

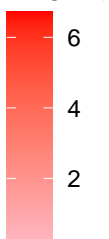

Gene number

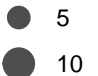

Supplement: Supplement 11 — Supplemental Figure 11. KEGG functional analysis results of influenza vaccines. Any pathway listed shows up as one of the top 10 most significant KEGG pathways for one of the nine gene sets. [file media-11.pdf]
