# Supplementary material for: SEA CDM: Study-Experiment-Assay Common Data Model and Databases for Cross-Domain Data Integration and Analysis
Source: bioRxiv. 2025 Aug 28:2025.08.26.671804. Preprint. [Version 1] doi: 10.1101/2025.08.26.671804 (PMC12407955; doi:10.1101/2025.08.26.671804)
Supplement: Supplement 12 — Supplemental Figure 12. ImmPort to SEA-CDM format modeling. A simplified mapping of key tables of ImmPort to SEA-CDM format. SEA-CDM foreign IDs require information that is found in linking tables to consolidate data (i.e., SEA-CDM Sample requires data loaded from the “Biosample”, “ControlSample”, “ExpSample” core tables and “Biosample-2-Expsample”. Additionally, information related to the SEA-CDM Sample’s Organism would require the use of the “Biosample-2-Subject” table. The original connections showing each table were taken from the ImmPort website. [file media-12.pdf]

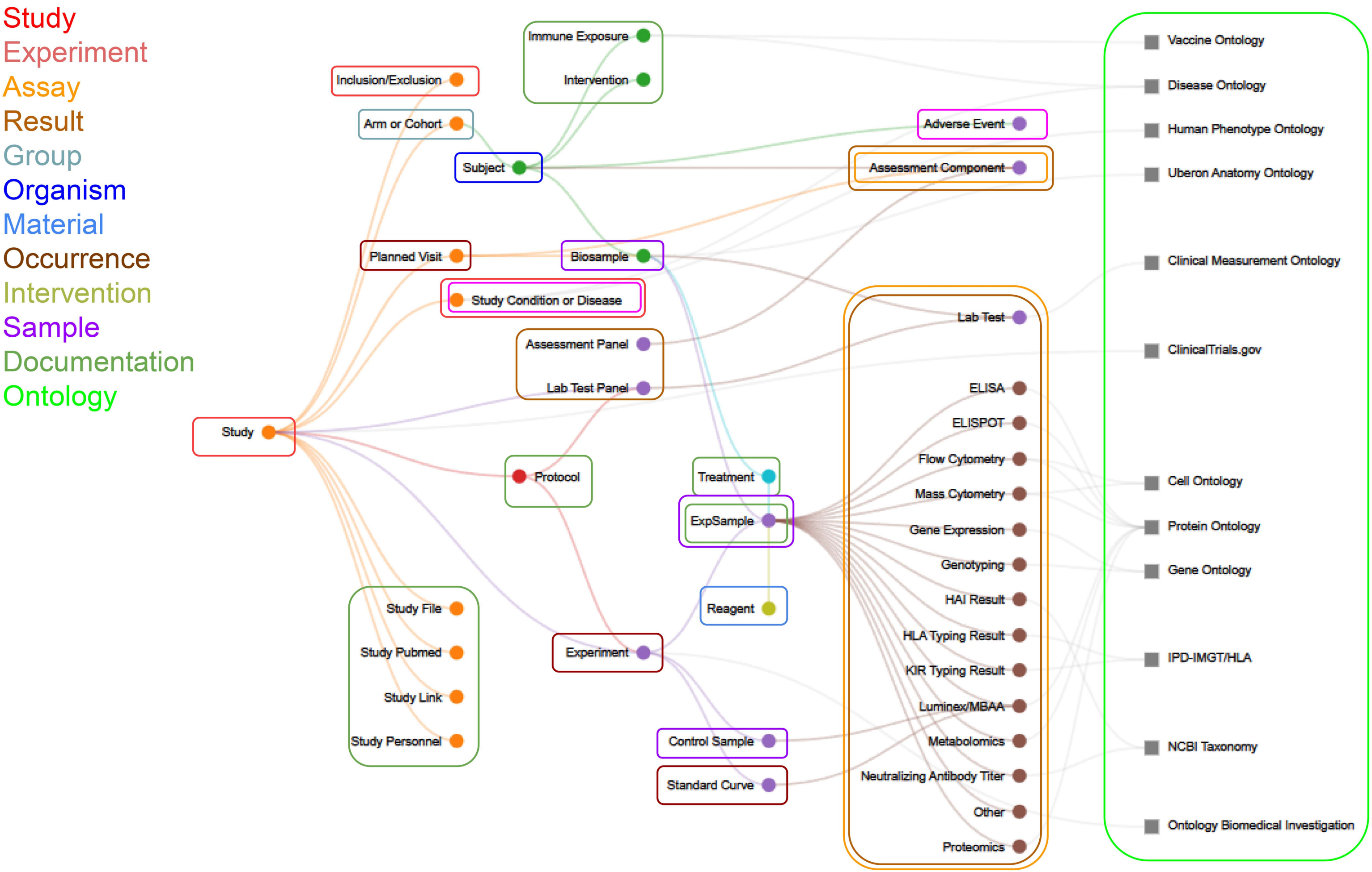

Study
Experiment
Assay
Result
Group
Organism
Material
Occurrence
Intervention
Sample
Documentation
Ontology
Immune Exposure
Intervention
Inclusion/Exclusion
Arm or Cohort
Subject
Adverse Event
Assessment Component
Planned Visit
Biosample
Study Condition or Disease
Assessment Panel
Lab Test Panel
Lab Test
Study
Protocol
Treatment
ExpSample
Reagent
Experiment
Control Sample
Standard Curve
Study File
Study Pubmed
Study Link
Study Personnel
ELISA
ELISPOT
Flow Cytometry
Mass Cytometry
Gene Expression
Genotyping
HAI Result
HLA Typing Result
KIR Typing Result
Luminex/MBAA
Metabolomics
Neutralizing Antibody Titer
Other
Proteomics
Vaccine Ontology
Disease Ontology
Human Phenotype Ontology
Uberon Anatomy Ontology
Clinical Measurement Ontology
ClinicalTrials.gov
Cell Ontology
Protein Ontology
Gene Ontology
IPD-IMGT/HLA
NCBI Taxonomy
Ontology Biomedical Investigation
