# Supplementary material for: SEA CDM: Study-Experiment-Assay Common Data Model and Databases for Cross-Domain Data Integration and Analysis
Source: bioRxiv. 2025 Aug 28:2025.08.26.671804. Preprint. [Version 1] doi: 10.1101/2025.08.26.671804 (PMC12407955; doi:10.1101/2025.08.26.671804)
Supplement: Supplement 13 [file media-13.docx]

**Supplemental Figures.**

**
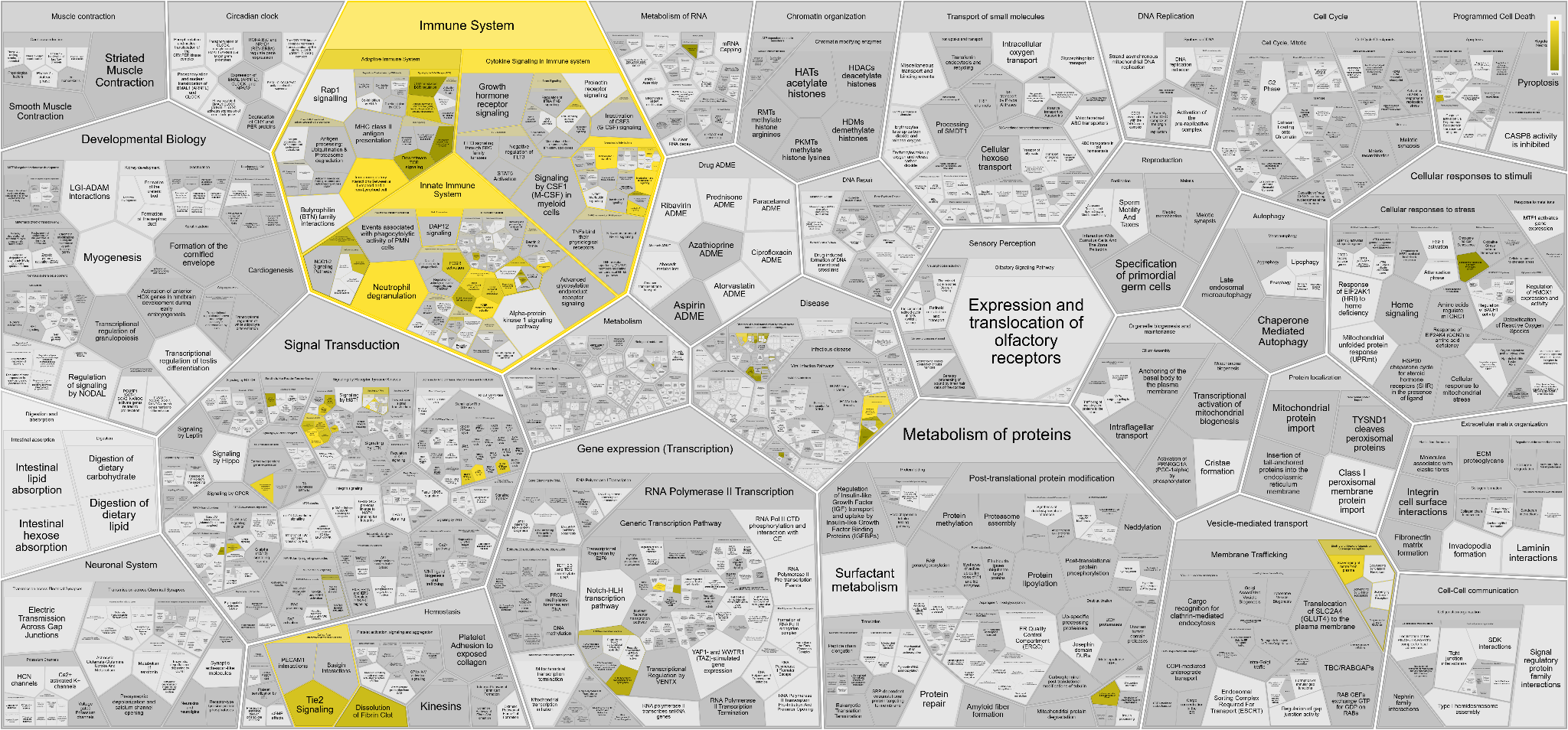
**

**Supplemental Figure 1. Full Reacfome representation of Reactome pathways stimulated by all Influenza vaccines in all-sex human subjects.** Gene set enrichment values can be found as part of Supplemental File 3.

**
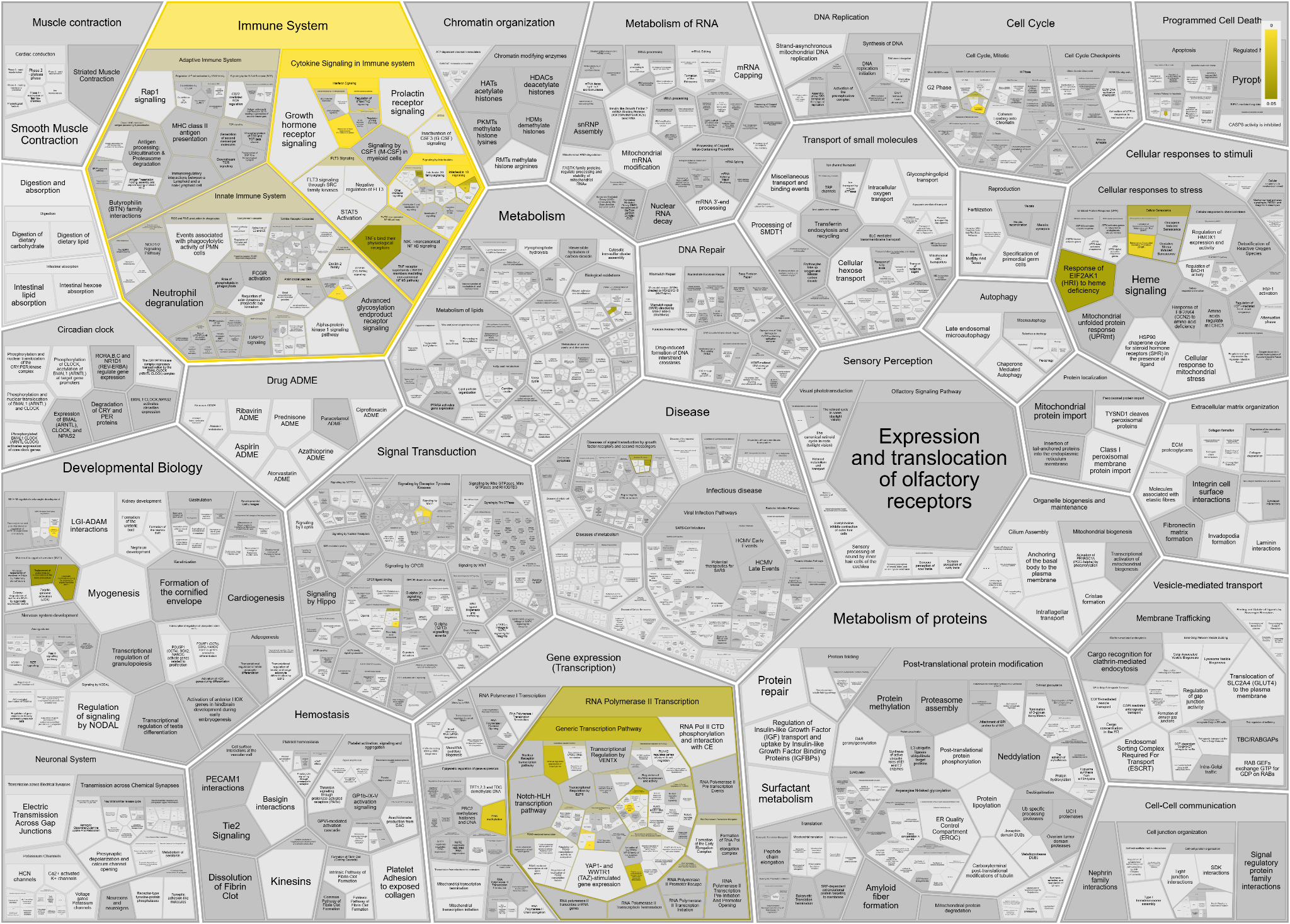
**

**Supplemental Figure 2. Full Reacfome representation of Reactome pathways stimulated by all Influenza vaccines in female human subjects.** Gene set enrichment values can be found as part of Supplemental File 3.

**
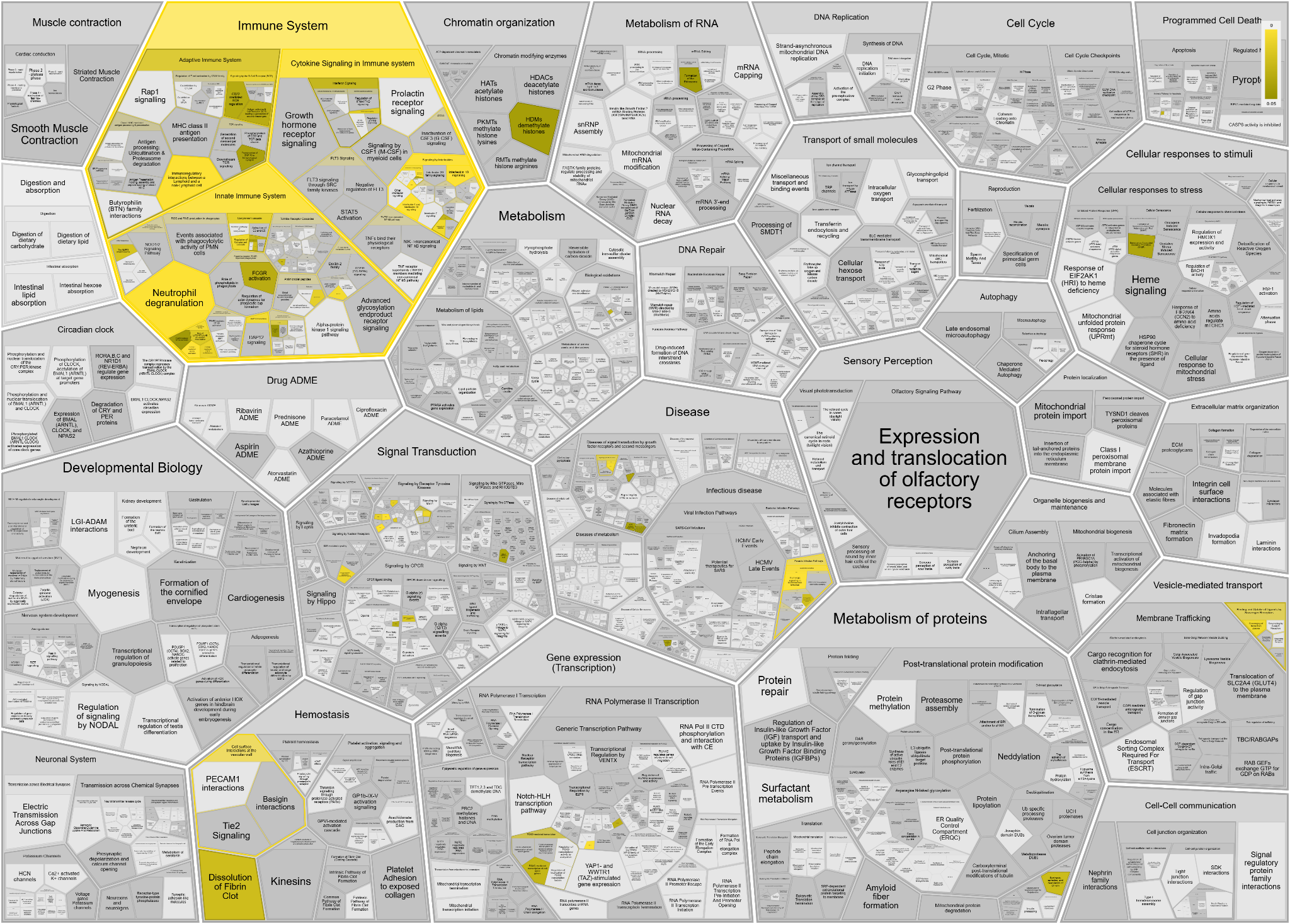
**

**Supplemental Figure 3. Full Reacfome representation of Reactome pathways stimulated by all Influenza vaccines in male human subjects.** Gene set enrichment values can be found as part of Supplemental File 3.

**
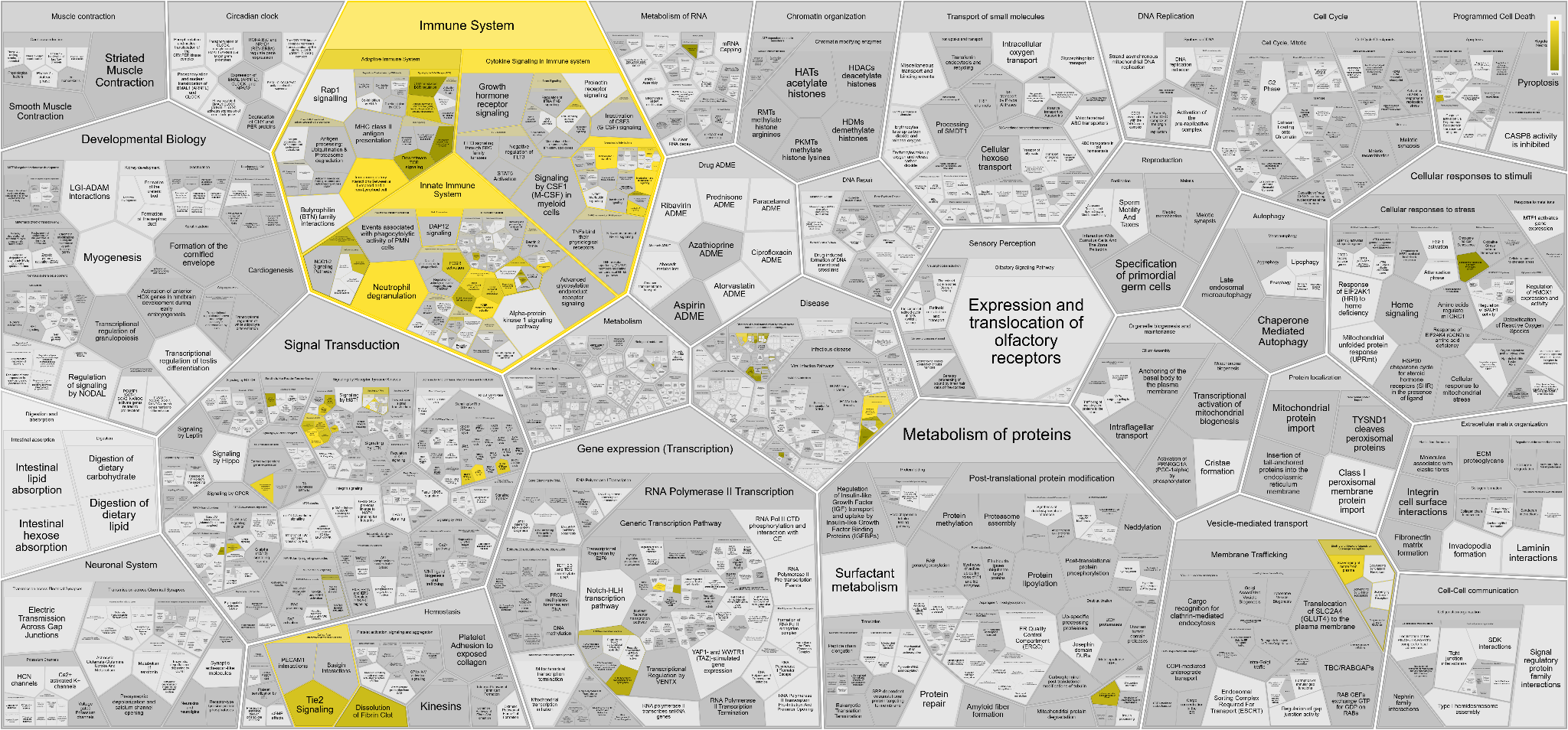
**

**Supplemental Figure 4. Full Reacfome representation of Reactome pathways stimulated by live attenuated Influenza vaccines in all sexes.** Gene set enrichment values can be found as part of Supplemental File 3.

**-
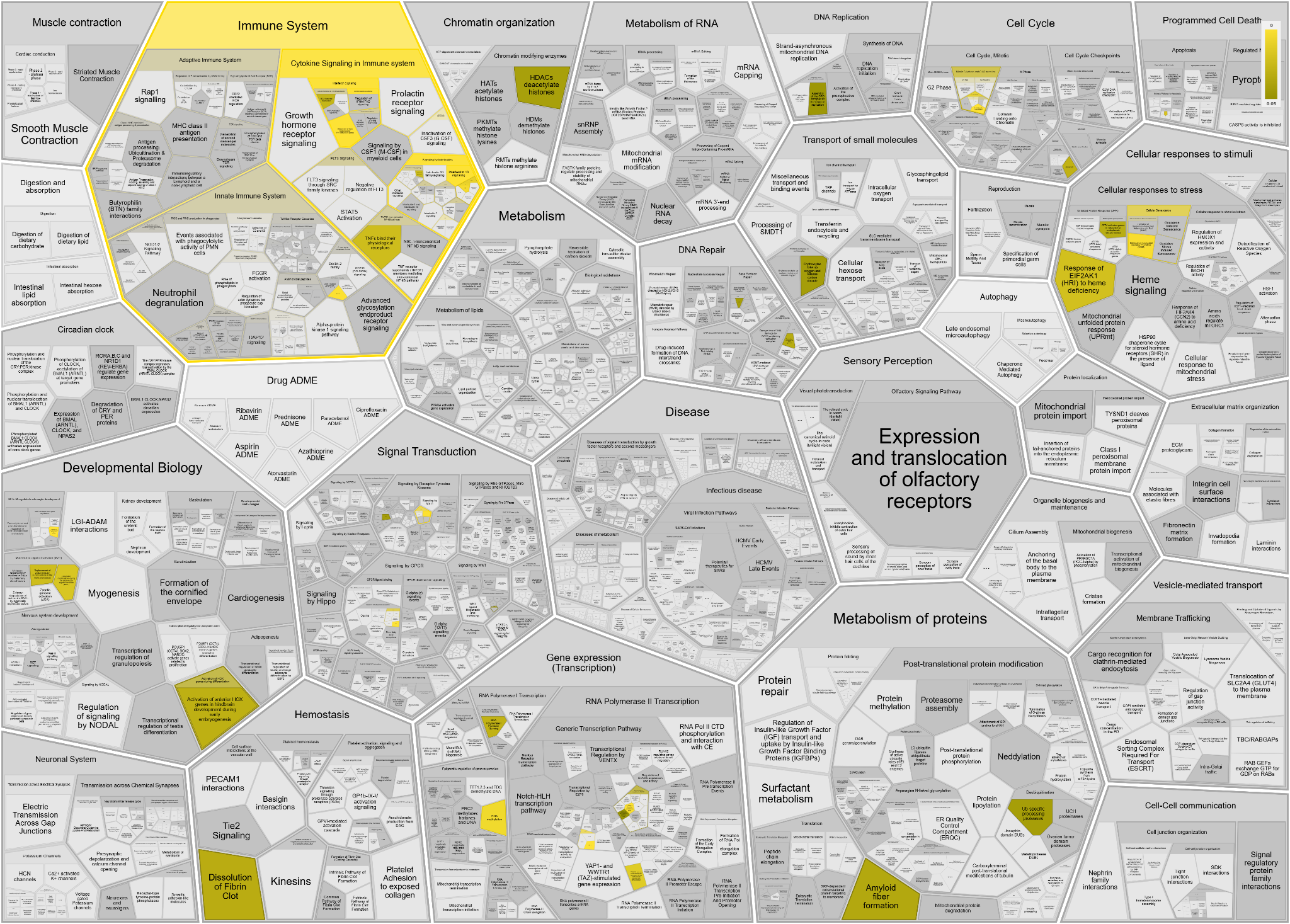
**

**Supplemental Figure 5. Full Reacfome representation of Reactome pathways stimulated by all Influenza and live attenuated Influenza vaccines in female human subjects.** Gene set enrichment values can be found as part of Supplemental File 3.

**
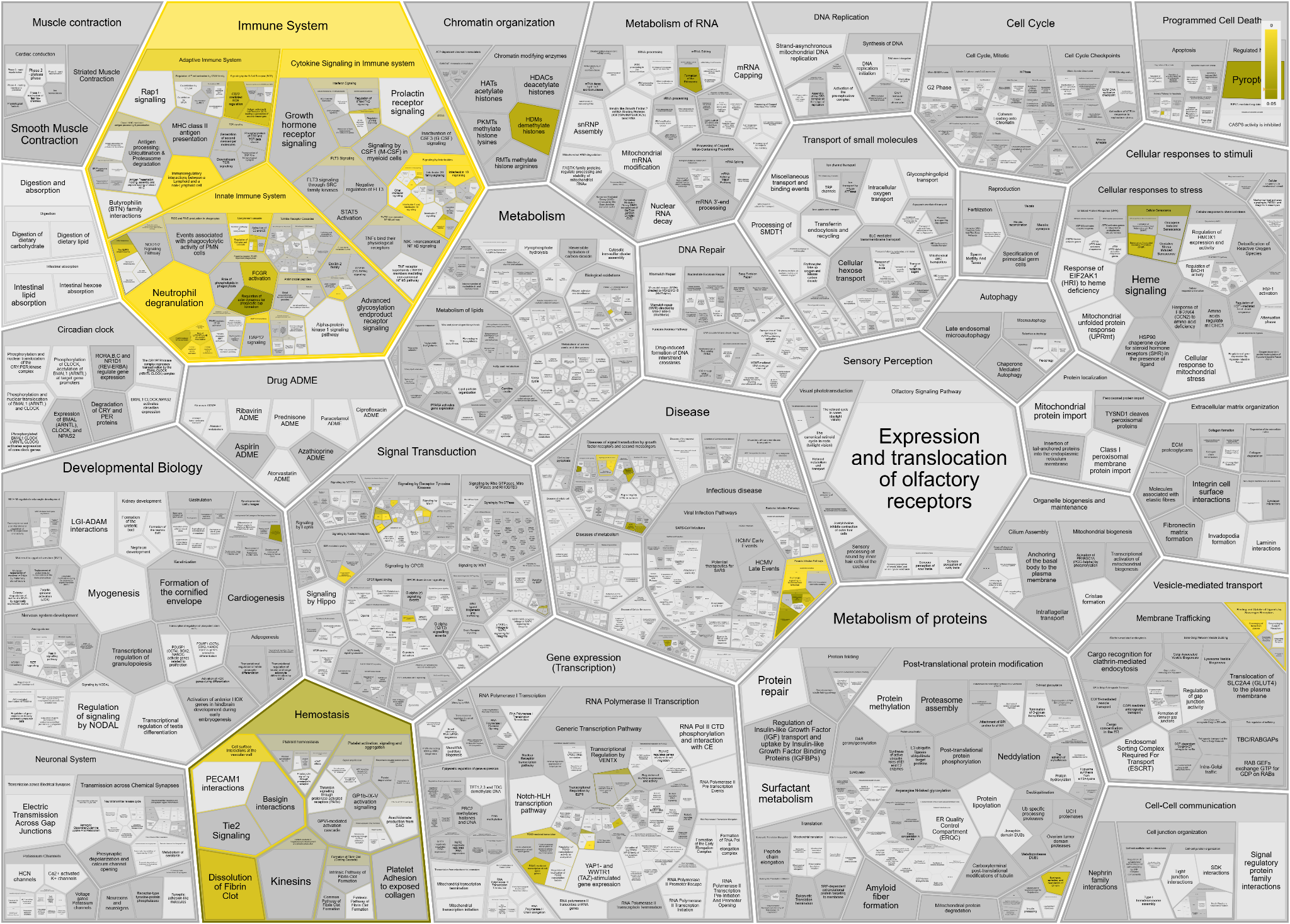
**

**Supplemental Figure 6. Full Reacfome representation of Reactome pathways stimulated by live attenuated Influenza vaccines in male human subjects.** Gene set enrichment values can be found as part of Supplemental File 3.

**
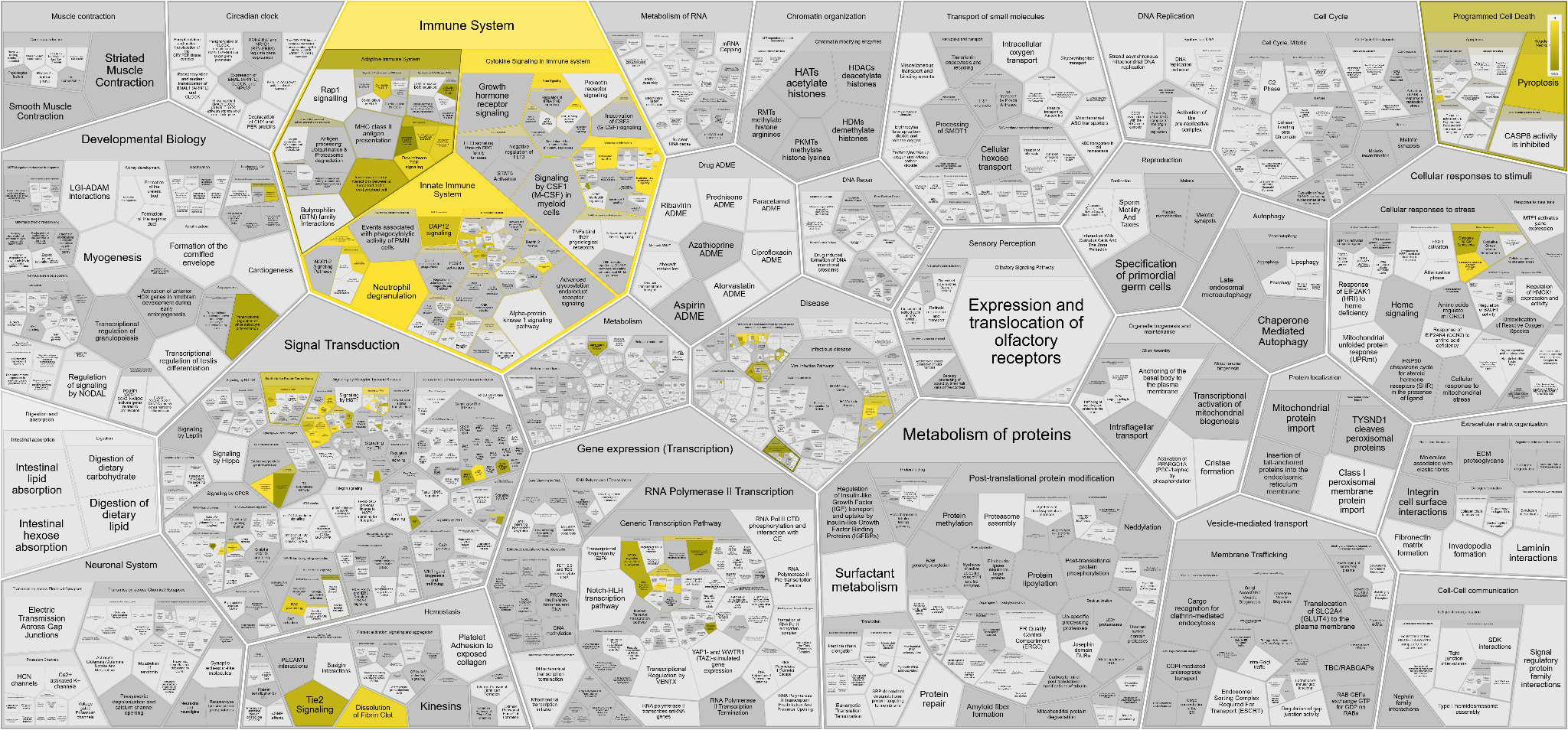
**

**Supplemental Figure 7. Full Reacfome representation of Reactome pathways stimulated by trivalent inactivated Influenza vaccines in all-sexes.** Gene set enrichment values can be found as part of Supplemental File 3.

**
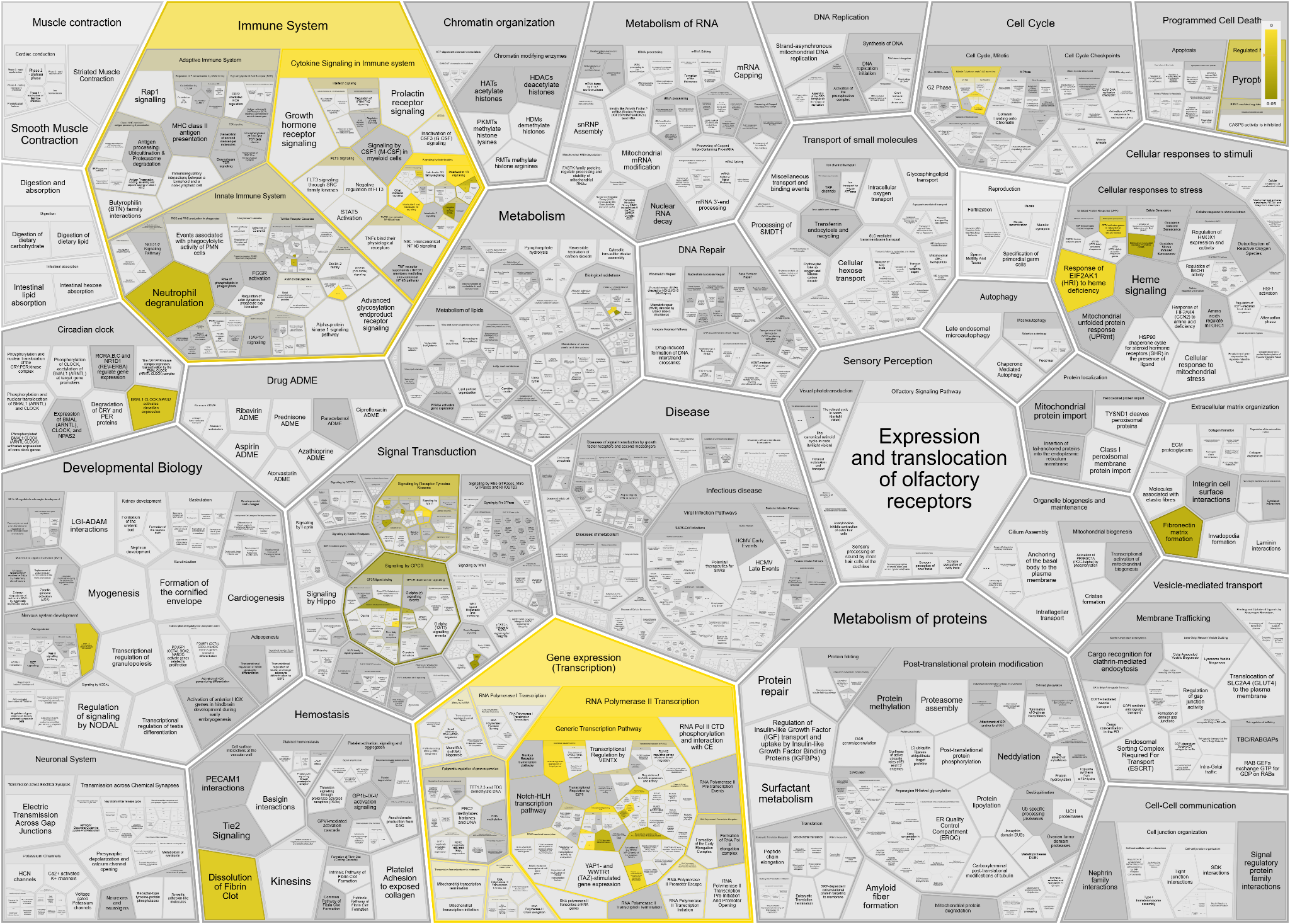
**

**Supplemental Figure 8. Full Reacfome representation of Reactome pathways stimulated by trivalent inactivated Influenza vaccines in female human subjects.** Gene set enrichment values can be found as part of Supplemental File 3.

**
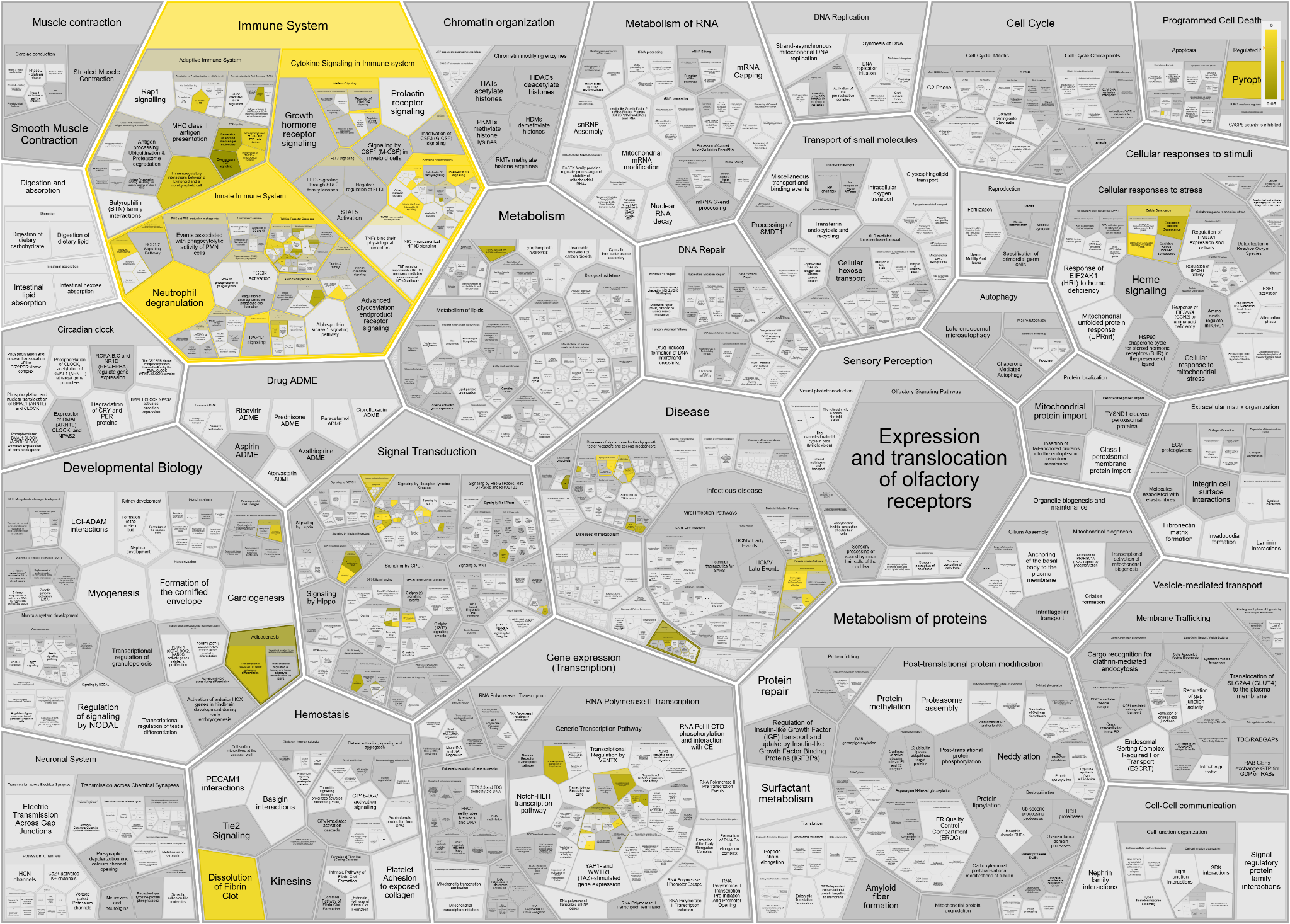
**

**Supplemental Figure 9. Full Reacfome representation of Reactome pathways stimulated by trivalent inactivated Influenza vaccines in male human subjects.** Gene set enrichment values can be found as part of Supplemental File 3.


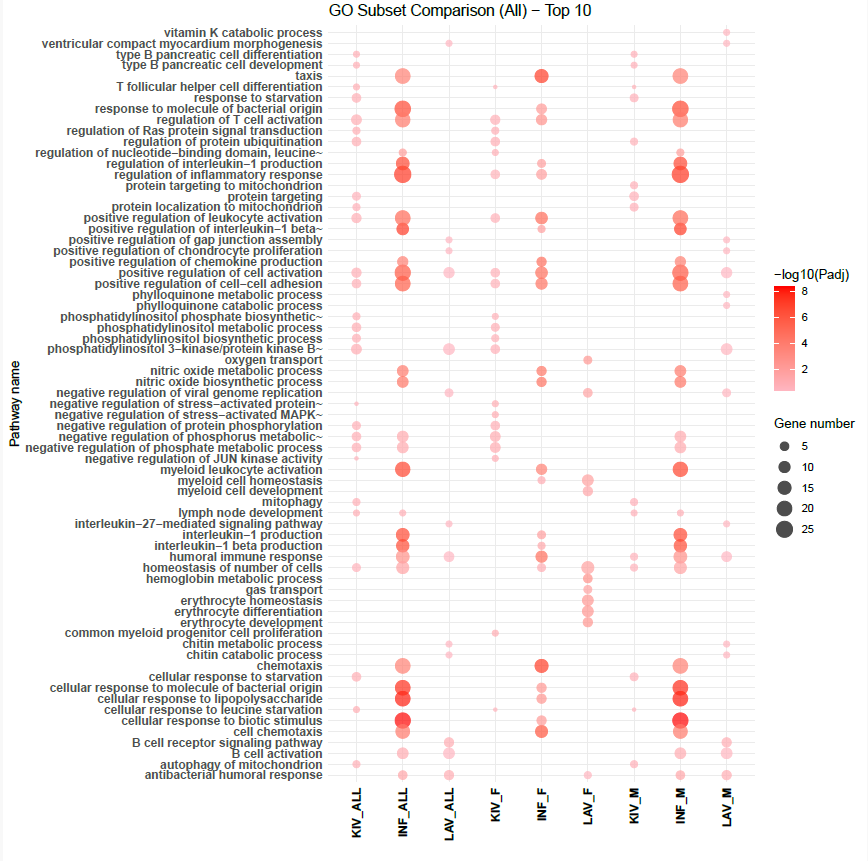


**Supplemental Figure 10. GO functional analysis results of influenza vaccines.** Any pathway listed shows up as one of the top-10 most significant GO pathways for one of the nine gene sets.


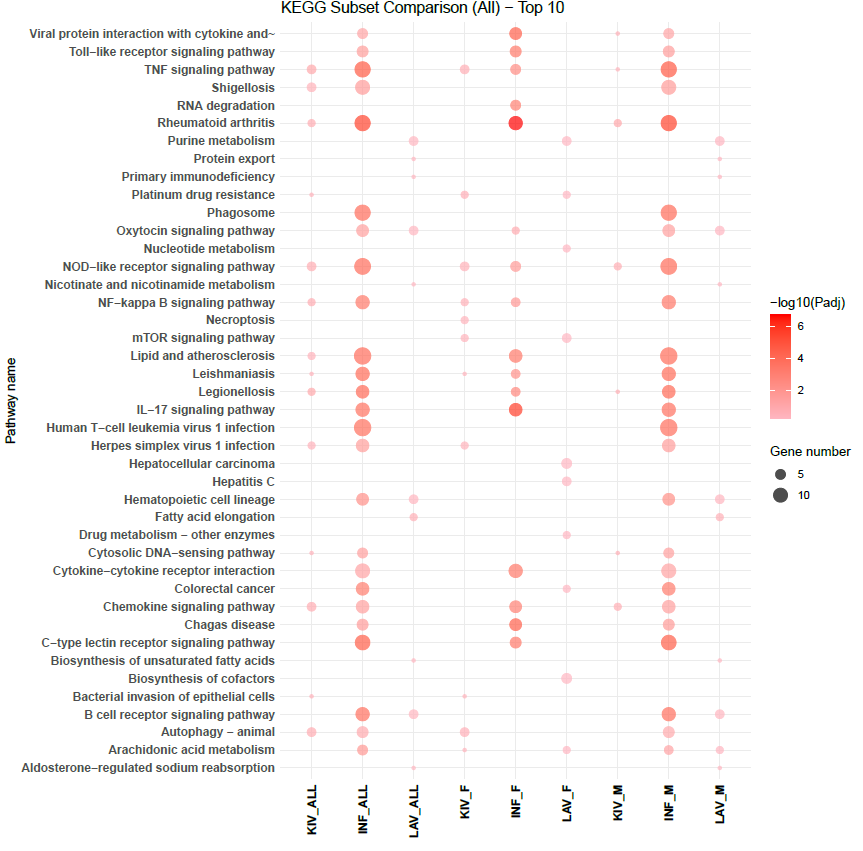


**Supplemental Figure 11. KEGG functional analysis results of influenza vaccines.** Any pathway listed shows up as one of the top-10 most significant KEGG pathways for one of the nine gene sets.


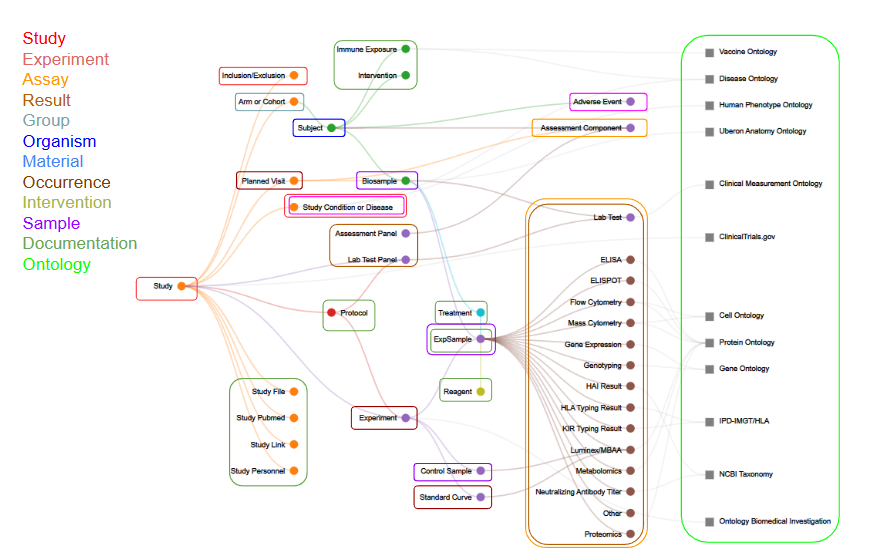


**Supplemental Figure 12. ImmPort to SEA-CDM format modeling.** A simplified mapping of key tables of ImmPort to SEA-CDM format. SEA-CDM foreign ids require information that are found in linking tables to consolidate data (i.e. SEA-CDM Sample requires data loaded from the “Biosample”, “ControlSample”, “ExpSample” core tables and “Biosample-2-Expsample”. Additionally, information related to SEA-CDM Sample’s Organism would require use of the “Biosample-2-Subject” table. The original connections showing each table was taken from the ImmPort website.
