# Supplementary material for: SEA CDM: Study-Experiment-Assay Common Data Model and Databases for Cross-Domain Data Integration and Analysis
Source: bioRxiv. 2025 Aug 28:2025.08.26.671804. Preprint. [Version 1] doi: 10.1101/2025.08.26.671804 (PMC12407955; doi:10.1101/2025.08.26.671804)
Supplement: Supplement 1 — Supplemental Figure 1. Full Reactome representation of Reactome pathways stimulated by all Influenza vaccines in all-sex human subjects. Gene set enrichment values can be found as part of Supplemental File 2. [file media-1.pdf]

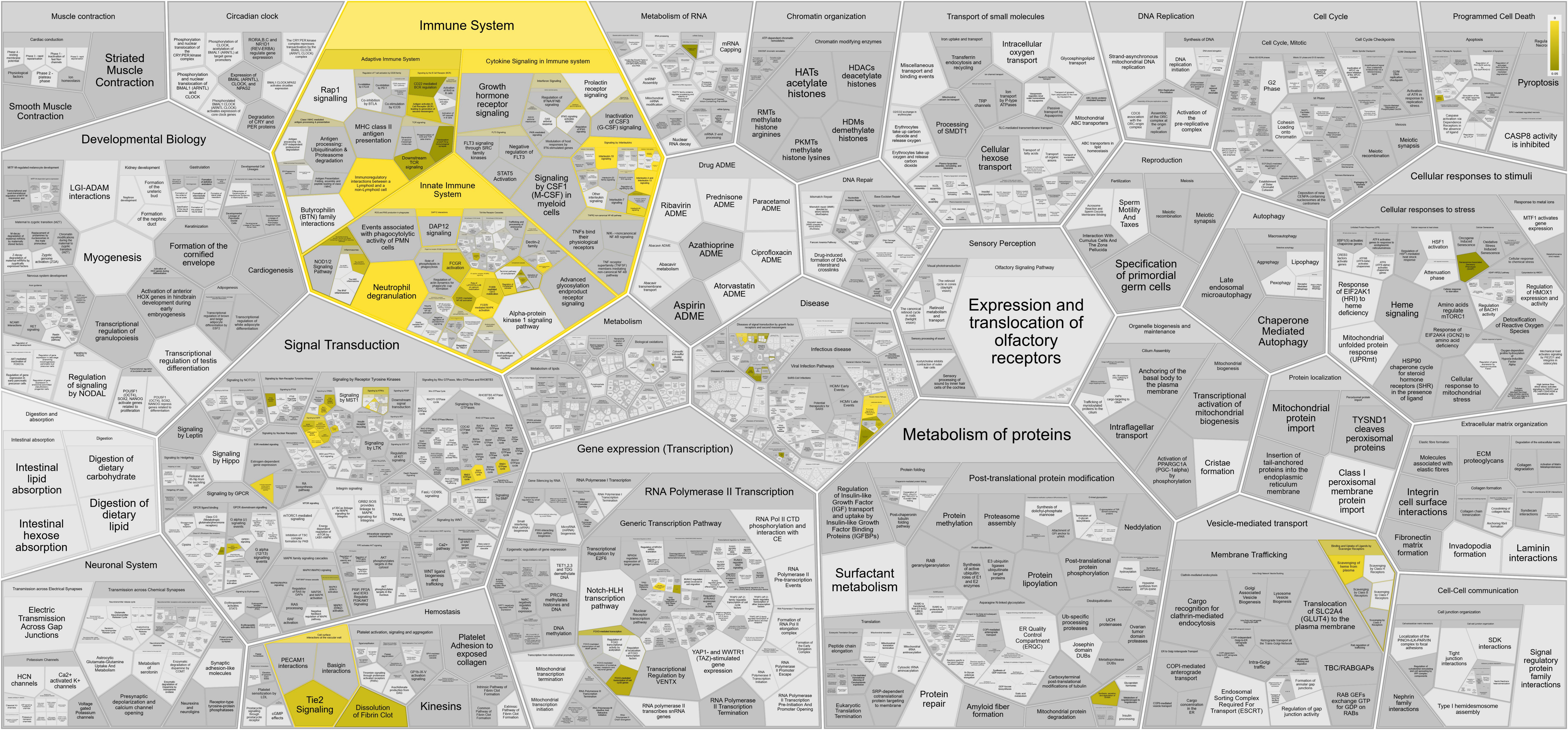
Muscle contraction
Striated Muscle Contraction
Smooth Muscle Contraction
Circadian clock
Immune System
Adaptive Immune System
Cytokine Signaling in Immune system
Rap1 signalling
MHC class II antigen presentation
Antigen processing: Ubiquitination & Proteasome degradation
Growth hormone receptor signaling
Prolactin receptor signaling
Inactivation of CSF3 (G-CSF) signaling
Innate Immune System
Butyrophilin (BTN) family interactions
Events associated with phagocytolytic activity of PMN cells
DAP12 signaling
Neutrophil degranulation
Signaling by CSF1 (M-CSF) in myeloid cells
STAT5 Activation
Advanced glycosylation endproduct receptor signaling
Alpha-protein kinase 1 signaling pathway
TNFs bind their physiological receptors
Metabolism of RNA
mRNA Capping
Chromatin organization
HATs acetylate histones
HDACs deacetylate histones
RMTs methylate histone arginines
PKMTs methylate histone lysines
HDMs demethylate histones
Transport of small molecules
Intracellular oxygen transport
Cellular hexose transport
Processing of SMDT1
DNA Replication
Cell Cycle
G2 Phase
Programmed Cell Death
Pyroptosis
CASP8 activity is inhibited
Developmental Biology
LGI-ADAM interactions
Myogenesis
Formation of the cornified envelope
Cardiogenesis
Regulation of signaling by NODAL
Transcriptional regulation of granulopoiesis
Transcriptional regulation of testis differentiation
Drug ADME
Ribavirin ADME
Prednisone ADME
Paracetamol ADME
Azathioprine ADME
Ciprofloxacin ADME
Atorvastatin ADME
Aspirin ADME
DNA Repair
Reproduction
Sperm Motility And Taxes
Specification of primordial germ cells
Cellular responses to stimuli
Cellular responses to stress
Autophagy
Chaperone Mediated Autophagy
Heme signaling
Sensory Perception
Expression and translocation of olfactory receptors
Disease
Metabolism
Signal Transduction
Gene expression (Transcription)
RNA Polymerase II Transcription
Generic Transcription Pathway
Metabolism of proteins
Post-translational protein modification
Surfactant metabolism
Protein localization
Mitochondrial protein import
TYSND1 cleaves peroxisomal proteins
Class I peroxisomal membrane protein import
Extracellular matrix organization
Integrin cell surface interactions
Laminin interactions
Vesicle-mediated transport
Membrane Trafficking
Cell-Cell communication
Digestion and absorption
Intestinal lipid absorption
Intestinal hexose absorption
Digestion of dietary carbohydrate
Digestion of dietary lipid
Neuronal System
Electric Transmission Across Gap Junctions
Hemostasis
Platelet Adhesion to exposed collagen
Tie2 Signaling
Kinesins
Dissolution of Fibrin Clot
Notch-HLH transcription pathway
Protein repair
Amyloid fiber formation
TBC/RABGAPs
Signal regulatory protein family interactions
