# Supplementary material for: SEA CDM: Study-Experiment-Assay Common Data Model and Databases for Cross-Domain Data Integration and Analysis
Source: bioRxiv. 2025 Aug 28:2025.08.26.671804. Preprint. [Version 1] doi: 10.1101/2025.08.26.671804 (PMC12407955; doi:10.1101/2025.08.26.671804)
Supplement: Supplement 6 — Supplemental Figure 6. Full Reactome representation of Reactome pathways stimulated by live attenuated Influenza vaccines in male human subjects. Gene set enrichment values can be found as part of Supplemental File 2. [file media-6.pdf]

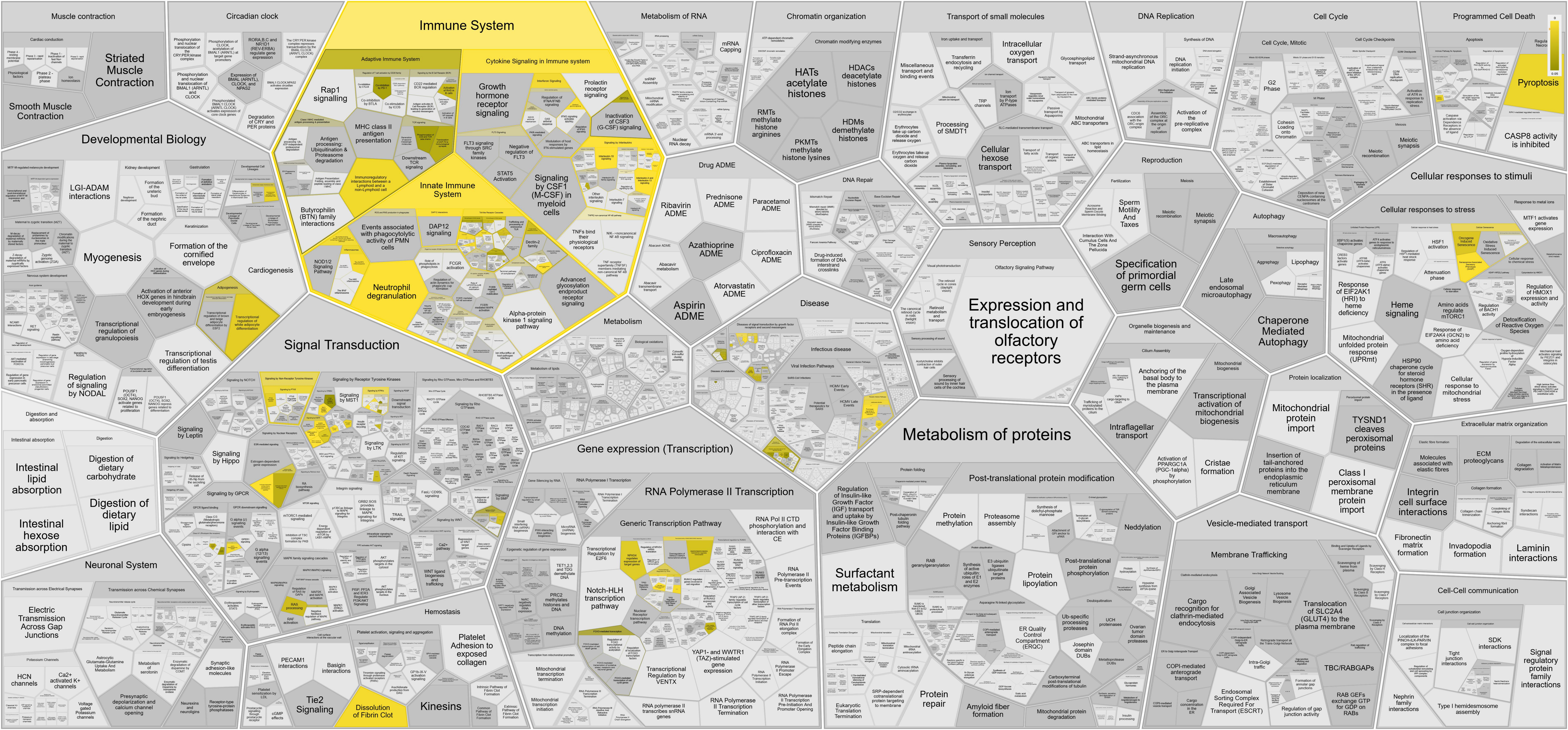

Muscle contraction
Striated Muscle Contraction
Smooth Muscle Contraction
Circadian clock
Immune System
Adaptive Immune System
Cytokine Signaling in Immune system
Rap1 signalling
Growth hormone receptor signaling
Prolactin receptor signaling
MHC class II antigen presentation
Antigen processing: Ubiquitination & Proteasome degradation
Inactivation of CSF3 (G-CSF) signaling
FLT3 signaling through SRC family kinases
Negative regulation of FLT3
Innate Immune System
STAT5 Activation
Signaling by CSF1 (M-CSF) in myeloid cells
Butyrophilin (BTN) family interactions
Events associated with phagocytolytic activity of PMN cells
DAP12 signaling
TNFs bind their physiological receptors
NOD1/2 Signaling Pathway
FCGR activation
Dectin-2 family
Neutrophil degranulation
Advanced glycosylation endproduct receptor signaling
Alpha-protein kinase 1 signaling pathway
Metabolism of RNA
mRNA Capping
Nuclear RNA decay
Chromatin organization
Chromatin modifying enzymes
HATs acetylate histones
HDACs deacetylate histones
RMTs methylate histone arginines
HDMs demethylate histones
PKMTs methylate histone lysines
Transport of small molecules
Intracellular oxygen transport
Miscellaneous transport and binding events
Transferrin endocytosis and recycling
Glycosphingolipid transport
Processing of SMDT1
Mitochondrial ABC transporters
Cellular hexose transport
DNA Replication
Strand-asynchronous mitochondrial DNA replication
DNA replication initiation
Activation of the pre-replicative complex
Cell Cycle
Cell Cycle, Mitotic
G2 Phase
Cohesin Loading onto Chromatin
Meiotic synapsis
Meiotic recombination
Programmed Cell Death
Pyroptosis
CASP8 activity is inhibited
Developmental Biology
LGI-ADAM interactions
Myogenesis
Formation of the cornified envelope
Cardiogenesis
Activation of anterior HOX genes in hindbrain development during early embryogenesis
Transcriptional regulation of granulopoiesis
Transcriptional regulation of testis differentiation
Regulation of signaling by NODAL
Drug ADME
Ribavirin ADME
Prednisone ADME
Paracetamol ADME
Azathioprine ADME
Ciprofloxacin ADME
Abacavir metabolism
Atorvastatin ADME
Aspirin ADME
DNA Repair
Reproduction
Sperm Motility And Taxes
Meiotic synapsis
Specification of primordial germ cells
Autophagy
Late endosomal microautophagy
Chaperone Mediated Autophagy
Cellular responses to stimuli
Cellular responses to stress
MTF1 activates gene expression
HSF1 activation
Response of EIF2AK1 (HRI) to heme deficiency
Heme signaling
Amino acids regulate mTORC1
Regulation of HMOX1 expression and activity
Detoxification of Reactive Oxygen Species
Response of EIF2AK4 (GCN2) to amino acid deficiency
Mitochondrial unfolded protein response (UPRmt)
HSP90 chaperone cycle for steroid hormone receptors (SHR) in the presence of ligand
Cellular response to mitochondrial stress
Sensory Perception
Olfactory Signaling Pathway
Expression and translocation of olfactory receptors
Disease
Infectious disease
Metabolism
Signal Transduction
Signaling by Leptin
Signaling by Hippo
Signaling by MST1
Signaling by LTK
Signaling by GPCR
Digestion and absorption
Intestinal lipid absorption
Digestion of dietary carbohydrate
Digestion of dietary lipid
Intestinal hexose absorption
Neuronal System
Electric Transmission Across Gap Junctions
HCN channels
Ca2+ activated K+ channels
Presynaptic depolarization and calcium channel opening
Synaptic adhesion-like molecules
Hemostasis
Platelet Adhesion to exposed collagen
PECAM1 interactions
Basigin interactions
Tie2 Signaling
Dissolution of Fibrin Clot
Kinesins
Gene expression (Transcription)
RNA Polymerase II Transcription
Generic Transcription Pathway
RNA Pol II CTD phosphorylation and interaction with CE
Notch-HLH transcription pathway
RNA Polymerase II Pre-transcription Events
PRC2 methylates histones and DNA
DNA methylation
YAP1- and WWTR1 (TAZ)-stimulated gene expression
Transcriptional Regulation by VENTX
RNA polymerase II transcribes snRNA genes
RNA Polymerase II Transcription Termination
Metabolism of proteins
Protein localization
Mitochondrial protein import
TYSND1 cleaves peroxisomal proteins
Insertion of tail-anchored proteins into the endoplasmic reticulum membrane
Class I peroxisomal membrane protein import
Organelle biogenesis and maintenance
Anchoring of the basal body to the plasma membrane
Transcriptional activation of mitochondrial biogenesis
Intraflagellar transport
Cristae formation
Activation of PPARGC1A (PGC-1alpha) by phosphorylation
Extracellular matrix organization
Molecules associated with elastic fibres
ECM proteoglycans
Integrin cell surface interactions
Fibronectin matrix formation
Invadopodia formation
Laminin interactions
Post-translational protein modification
Regulation of Insulin-like Growth Factor (IGF) transport and uptake by Insulin-like Growth Factor Binding Proteins (IGFBPs)
Protein methylation
Proteasome assembly
Neddylation
Surfactant metabolism
Protein lipoylation
Post-translational protein phosphorylation
ER Quality Control Compartment (ERQC)
Ub-specific processing proteases
Josephin domain DUBs
UCH proteinases
Ovarian tumor domain proteases
Carboxyterminal post-translational modifications of tubulin
Amyloid fiber formation
Mitochondrial protein degradation
Protein repair
Translation
Peptide chain elongation
Eukaryotic Translation Termination
Vesicle-mediated transport
Membrane Trafficking
Cargo recognition for clathrin-mediated endocytosis
Translocation of SLC2A4 (GLUT4) to the plasma membrane
COPI-mediated anterograde transport
Endosomal Sorting Complex Required For Transport (ESCRT)
TBC/RABGAPs
RAB GEFs exchange GTP for GDP on RABs
Cell-Cell communication
SDK interactions
Signal regulatory protein family interactions
Nephrin family interactions
Type I hemidesmosome assembly
