# Supplementary material for: SEA CDM: Study-Experiment-Assay Common Data Model and Databases for Cross-Domain Data Integration and Analysis
Source: bioRxiv. 2025 Aug 28:2025.08.26.671804. Preprint. [Version 1] doi: 10.1101/2025.08.26.671804 (PMC12407955; doi:10.1101/2025.08.26.671804)
Supplement: Supplement 7 — Supplemental Figure 7. Full Reactome representation of Reactome pathways stimulated by trivalent inactivated Influenza vaccines in all-sexes. Gene set enrichment values can be found as part of Supplemental File 2. [file media-7.pdf]

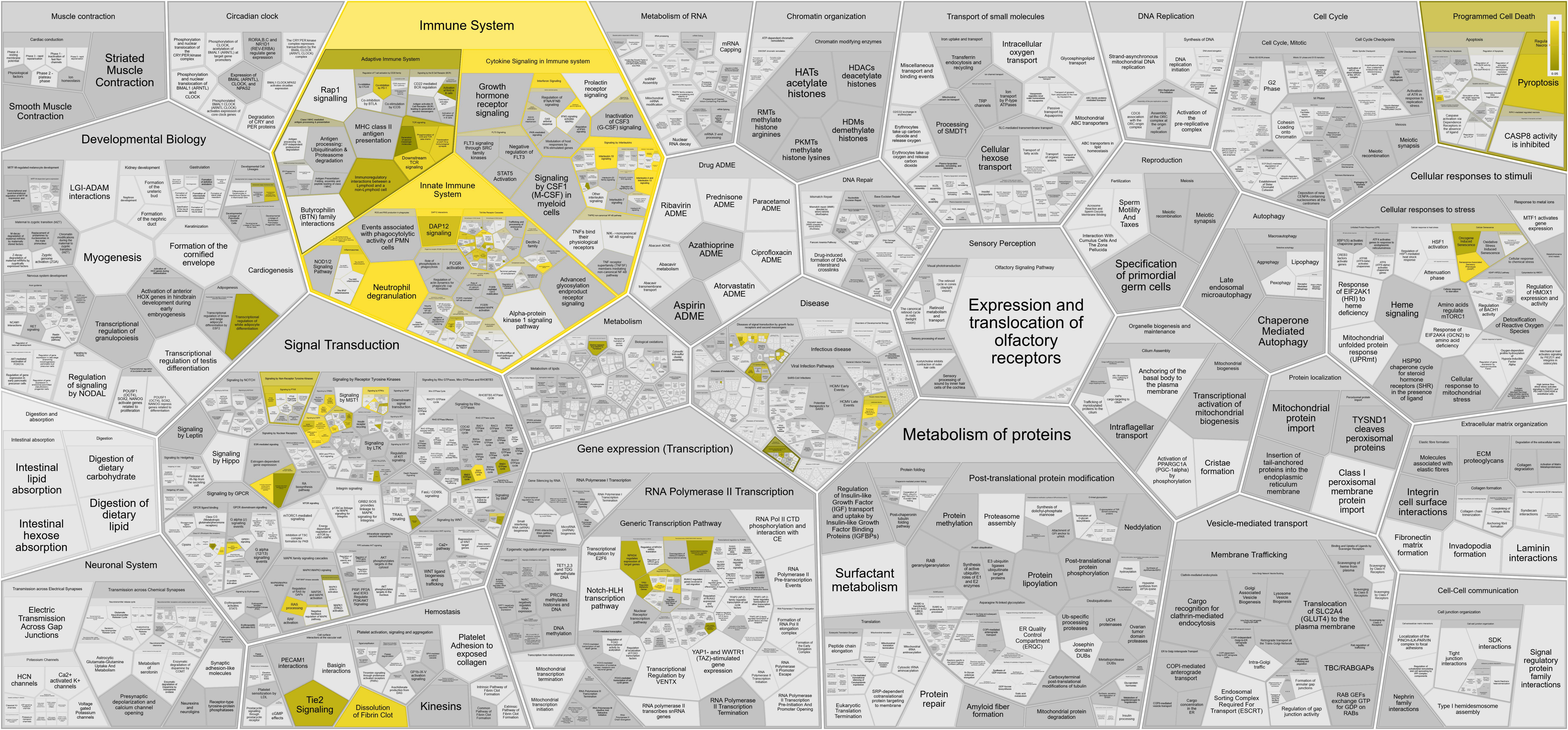

Muscle contraction
Striated Muscle Contraction
Smooth Muscle Contraction
Cardiac conduction
Circadian clock
Immune System
Adaptive Immune System
Cytokine Signaling in Immune system
Rap1 signaling
MHC class II antigen presentation
Antigen processing: Ubiquitination & Proteasome degradation
Growth hormone receptor signaling
Prolactin receptor signaling
Inactivation of CSF3 (G-CSF) signaling
FLT3 signaling through SRC family kinases
Negative regulation of FLT3
STAT5 Activation
Signaling by CSF1 (M-CSF) in myeloid cells
Innate Immune System
Butyrophilin (BTN) family interactions
Events associated with phagocytolytic activity of PMN cells
DAP12 signaling
NOD1/2 Signaling Pathway
Neutrophil degranulation
Advanced glycosylation endproduct receptor signaling
Alpha-protein kinase 1 signaling pathway
TNFs bind their physiological receptors
Metabolism of RNA
mRNA Capping
Nuclear RNA decay
Chromatin organization
Chromatin modifying enzymes
HATs acetylate histones
HDACs deacetylate histones
RMTs methylate histone arginines
HDMs demethylate histones
PKMTs methylate histone lysines
Transport of small molecules
Intracellular oxygen transport
Miscellaneous transport and binding events
Processing of SMDT1
Cellular hexose transport
Mitochondrial ABC transporters
DNA Replication
DNA replication initiation
Activation of the pre-replicative complex
Cohesin Loading onto Chromatin
Cell Cycle
Cell Cycle, Mitotic
G2 Phase
Meiotic synapsis
Programmed Cell Death
Pyroptosis
CASP8 activity is inhibited
Developmental Biology
LGI-ADAM interactions
Myogenesis
Formation of the cornified envelope
Cardiogenesis
Transcriptional regulation of granulopoiesis
Transcriptional regulation of testis differentiation
Regulation of signaling by NODAL
Drug ADME
Ribavirin ADME
Prednisone ADME
Paracetamol ADME
Azathioprine ADME
Ciprofloxacin ADME
Atorvastatin ADME
Aspirin ADME
DNA Repair
Disease
Infectious disease
Viral Infection Pathways
Sensory Perception
Olfactory Signaling Pathway
Expression and translocation of olfactory receptors
Reproduction
Specification of primordial germ cells
Meiotic synapsis
Autophagy
Chaperone Mediated Autophagy
Late endosomal microautophagy
Cellular responses to stimuli
Cellular responses to stress
MTF1 activates gene expression
Heme signaling
Response of EIF2AK1 (HRI) to heme deficiency
Mitochondrial unfolded protein response (UPRmt)
Cellular response to mitochondrial stress
Signal Transduction
Metabolism
Digestion and absorption
Intestinal lipid absorption
Digestion of dietary carbohydrate
Digestion of dietary lipid
Intestinal hexose absorption
Signaling by Leptin
Signaling by Hippo
Signaling by GPCR
Signaling by MST1
Signaling by LTK
Gene expression (Transcription)
RNA Polymerase II Transcription
Generic Transcription Pathway
RNA Pol II CTD phosphorylation and interaction with CE
Notch-HLH transcription pathway
YAP1- and WWTR1 (TAZ)-stimulated gene expression
Transcriptional Regulation by VENTX
Metabolism of proteins
Post-translational protein modification
Surfactant metabolism
Protein methylation
Proteasome assembly
Neddylation
Protein lipoylation
Post-translational protein phosphorylation
Protein repair
Amyloid fiber formation
Anchoring of the basal body to the plasma membrane
Intraflagellar transport
Transcriptional activation of mitochondrial biogenesis
Cristae formation
Protein localization
Mitochondrial protein import
TYSND1 cleaves peroxisomal proteins
Class I peroxisomal membrane protein import
Extracellular matrix organization
Integrin cell surface interactions
Fibronectin matrix formation
Invadopodia formation
Laminin interactions
Vesicle-mediated transport
Membrane Trafficking
Cargo recognition for clathrin-mediated endocytosis
Translocation of SLC2A4 (GLUT4) to the plasma membrane
TBC/RABGAPs
Cell-Cell communication
SDK interactions
Signal regulatory protein family interactions
Neuronal System
Electric Transmission Across Gap Junctions
Transmission across Chemical Synapses
HCN channels
Hemostasis
PECAM1 interactions
Tie2 Signaling
Dissolution of Fibrin Clot
Platelet Adhesion to exposed collagen
Kinesins
