# Supplementary material for: SEA CDM: Study-Experiment-Assay Common Data Model and Databases for Cross-Domain Data Integration and Analysis
Source: bioRxiv. 2025 Aug 28:2025.08.26.671804. Preprint. [Version 1] doi: 10.1101/2025.08.26.671804 (PMC12407955; doi:10.1101/2025.08.26.671804)
Supplement: Supplement 8 — Supplemental Figure 8. Full Reactome representation of Reactome pathways stimulated by trivalent inactivated Influenza vaccines in female human subjects. Gene set enrichment values can be found as part of Supplemental File 2. [file media-8.pdf]

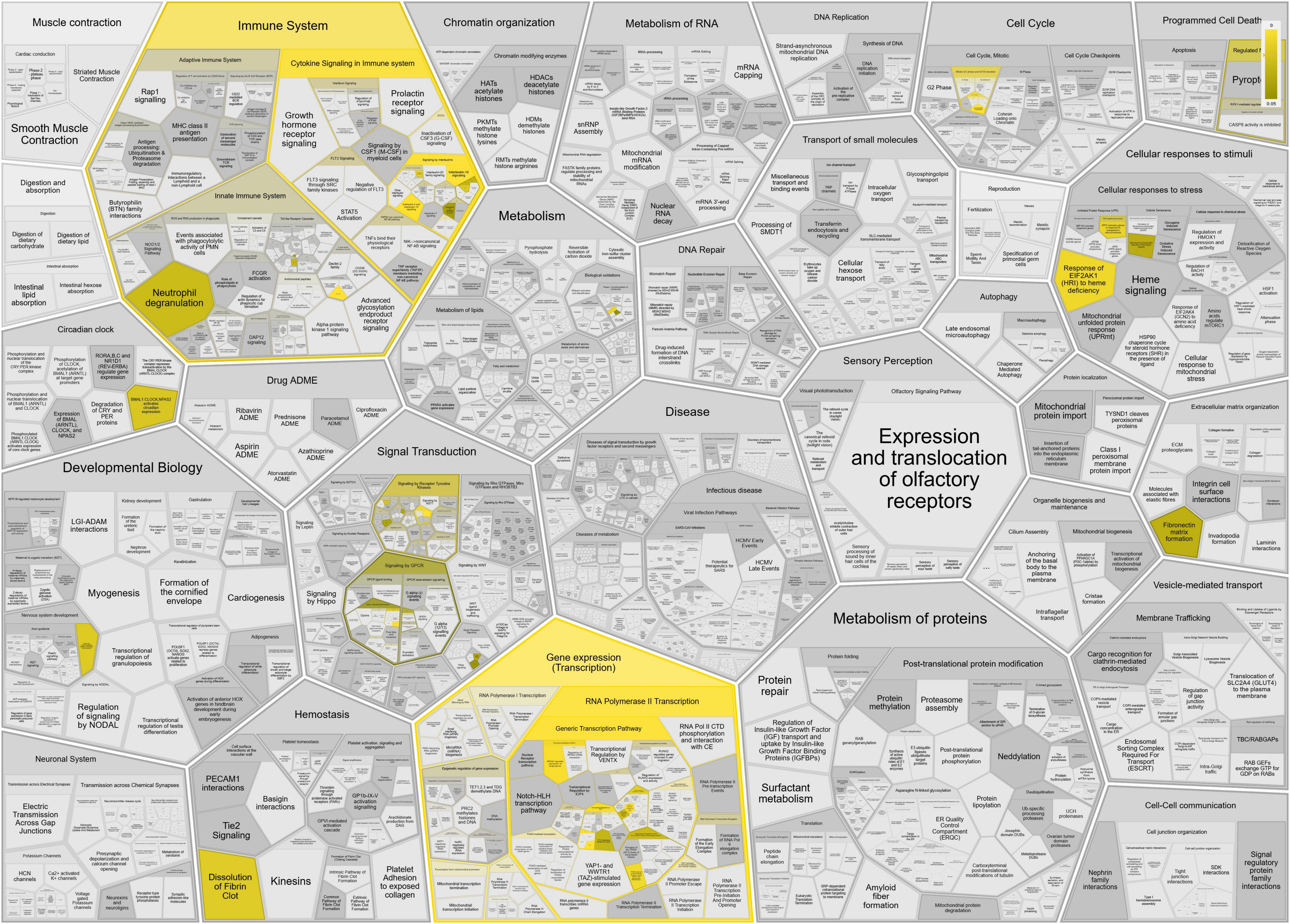

Muscle contraction
Cardiac conduction
Striated Muscle Contraction
Smooth Muscle Contraction
Digestion and absorption
Digestion
Digestion of dietary carbohydrate
Digestion of dietary lipid
Intestinal absorption
Intestinal lipid absorption
Intestinal hexose absorption
Immune System
Adaptive Immune System
Rap1 signalling
MHC class II antigen presentation
Antigen processing: Ubiquitination & Proteasome degradation
Butyrophilin (BTN) family interactions
Immunoregulatory interactions between a Lymphoid and a non-Lymphoid cell
Cytokine Signaling in Immune system
Growth hormone receptor signaling
Prolactin receptor signaling
Inactivation of CSF3 (G-CSF) signaling
Signaling by CSF1 (M-CSF) in myeloid cells
Signaling by Interleukins
FLT3 Signaling
FLT3 signaling through SRC family kinases
Negative regulation of FLT3
STAT5 Activation
TNFs bind their physiological receptors
NIK-->noncanonical NF-kB signaling
Innate Immune System
Events associated with phagocytolytic activity of PMN cells
NOD1/2 Signaling Pathway
FCGR activation
Neutrophil degranulation
Dectin-2 family
Advanced glycosylation endproduct receptor signaling
Alpha-protein kinase 1 signaling pathway
DAP12 signaling
Circadian clock
Phosphorylation of CLOCK, acetylation of BMAL1 (ARNTL) at target gene promoters
RORA,B,C and NR1D1 (REV-ERBA) regulate gene expression
Expression of BMAL (ARNTL), CLOCK, and NPAS2
Degradation of CRY and PER proteins
BMAL1:CLOCK,NPAS2 activates circadian expression
Chromatin organization
Chromatin modifying enzymes
HATs acetylate histones
HDACs deacetylate histones
PKMTs methylate histone lysines
HDMs demethylate histones
RMTs methylate histone arginines
Metabolism of RNA
mRNA Capping
snRNP Assembly
Mitochondrial mRNA modification
Nuclear RNA decay
mRNA 3'-end processing
DNA Replication
Strand-asynchronous mitochondrial DNA replication
Synthesis of DNA
DNA replication initiation
Activation of the pre-replicative complex
Cell Cycle
Cell Cycle, Mitotic
Cell Cycle Checkpoints
G2 Phase
Cohesin Loading onto Chromatin
Programmed Cell Death
Apoptosis
Regulated Necrosis
Pyroptosis
CASP8 activity is inhibited
Transport of small molecules
Miscellaneous transport and binding events
Intracellular oxygen transport
Glycosphingolipid transport
Transferrin endocytosis and recycling
Processing of SMDT1
Cellular hexose transport
Metabolism
Metabolism of lipids
DNA Repair
Drug-induced formation of DNA interstrand crosslinks
Reproduction
Fertilization
Specification of primordial germ cells
Autophagy
Late endosomal microautophagy
Chaperone Mediated Autophagy
Cellular responses to stimuli
Cellular responses to stress
Response of EIF2AK1 (HRI) to heme deficiency
Heme signaling
Mitochondrial unfolded protein response (UPRmt)
HSP90 chaperone cycle for steroid hormone receptors (SHR) in the presence of ligand
Cellular response to mitochondrial stress
Regulation of HMOX1 expression and activity
Detoxification of Reactive Oxygen Species
HSF1 activation
Amino acids regulate mTORC1
Sensory Perception
Visual phototransduction
Olfactory Signaling Pathway
Expression and translocation of olfactory receptors
Protein localization
Mitochondrial protein import
TYSND1 cleaves peroxisomal proteins
Class I peroxisomal membrane protein import
Extracellular matrix organization
ECM proteoglycans
Integrin cell surface interactions
Molecules associated with elastic fibres
Fibronectin matrix formation
Invadopodia formation
Laminin interactions
Drug ADME
Ribavirin ADME
Prednisone ADME
Paracetamol ADME
Ciprofloxacin ADME
Aspirin ADME
Azathioprine ADME
Atorvastatin ADME
Signal Transduction
Signaling by Receptor Tyrosine Kinases
Signaling by Rho GTPases, Miro GTPases and RHOBTB3
Signaling by GPCR
Signaling by Hippo
Signaling by WNT
Disease
Infectious disease
Viral Infection Pathways
HCMV Early Events
HCMV Late Events
Potential therapeutics for SARS
Diseases of metabolism
Developmental Biology
LGI-ADAM interactions
Myogenesis
Formation of the cornified envelope
Cardiogenesis
Nervous system development
Transcriptional regulation of granulopoiesis
Regulation of signaling by NODAL
Transcriptional regulation of testis differentiation
Activation of anterior HOX genes in hindbrain development during early embryogenesis
Neuronal System
Transmission across Chemical Synapses
Electric Transmission Across Gap Junctions
Potassium Channels
HCN channels
Ca2+ activated K+ channels
Voltage gated Potassium channels
Neurexins and neuroligins
Hemostasis
PECAM1 interactions
Basigin interactions
Tie2 Signaling
GP1b-IX-V activation signalling
GPVI-mediated activation cascade
Dissolution of Fibrin Clot
Kinesins
Platelet Adhesion to exposed collagen
Gene expression (Transcription)
RNA Polymerase I Transcription
RNA Polymerase II Transcription
Generic Transcription Pathway
RNA Pol II CTD phosphorylation and interaction with CE
Notch-HLH transcription pathway
Transcriptional Regulation by VENTX
TET1,2,3 and TDG demethylate DNA
PRC2 methylates histones and DNA
YAP1- and WWTR1 (TAZ)-stimulated gene expression
Metabolism of proteins
Protein folding
Post-translational protein modification
Protein repair
Protein methylation
Proteasome assembly
Neddylation
Regulation of Insulin-like Growth Factor (IGF) transport and uptake by Insulin-like Growth Factor Binding Proteins (IGFBPs)
Surfactant metabolism
Post-translational protein phosphorylation
Protein lipoylation
ER Quality Control Compartment (ERQC)
Translation
Peptide chain elongation
Amyloid fiber formation
Carboxyterminal post-translational modifications of tubulin
Mitochondrial protein degradation
Organelle biogenesis and maintenance
Cilium Assembly
Mitochondrial biogenesis
Anchoring of the basal body to the plasma membrane
Intraflagellar transport
Cristae formation
Vesicle-mediated transport
Membrane Trafficking
Cargo recognition for clathrin-mediated endocytosis
Translocation of SLC2A4 (GLUT4) to the plasma membrane
Regulation of gap junction activity
Endosomal Sorting Complex Required For Transport (ESCRT)
TBC/RABGAPs
RAB GEFs exchange GTP for GDP on RABs
Cell-Cell communication
Cell junction organization
Nephrin family interactions
Tight junction interactions
SDK interactions
Signal regulatory protein family interactions
0
0.05
